# Acute lung injury: an integrated view of molecular drivers, immune dysregulation, and emerging therapeutic strategies

**DOI:** 10.3389/fimmu.2026.1937966

**Published:** 2026-10-09

**Authors:** Aihua Wang, Haizhao Liu, Chengfen Yin, Yawen Song, Yue Jin, Lei Xu, Siwei Wang, Jiaxin Li, Xiping Guo, Yongli Ma, Yutong Jin, Wei Liu, Ximo Wang

**Affiliations:** 1 Central Hospital, Tianjin University, Tianjin, China; 2 Tianjin Key Laboratory of Extracorporeal Life Support for Critical Diseases, Tianjin, China; 3 Tianjin Artificial Cell Engineering Technology Research Center, Tianjin, China; 4 Tianjin First Central Hospital, Affiliated Hospital of Nankai University, Tianjin, China; 5 Tianjin Institute of Integrated Traditional Chinese and Western Medicine for Critical Care and Emergency Medicine, Tianjin, China; 6 First Teaching Hospital of Tianjin University of Traditional Chinese Medicine, Tianjin, China; 7 National Clinical Research Center for Chinese Medicine Acupuncture and Moxibustion, Tianjin, China

**Keywords:** acute lung injury, acute respiratory distress syndrome, epigenetic regulation, pathogenesis, signaling pathway, treatment

## Abstract

Acute lung injury (ALI, a term widely used in preclinical animal studies, while the clinical definition of ALI was eliminated in the Berlin criteria) and its severe human clinical counterpart, acute respiratory distress syndrome (ARDS), constitute life-threatening clinical syndromes characterized by diffuse alveolar damage, pulmonary edema, and severe hypoxemia. These conditions are associated with dismal mortality rates and significantly impair patient survival and recovery. Over the past decades, advancements in critical care medicine and molecular biology have deepened our understanding of the pathological mechanisms underlying ALI and ARDS. Aberrantly activated signaling pathways and molecular and cellular events have emerged as key elements to decipher core pathogenic processes, identify early biomarkers, and develop novel therapeutic strategies. This review aims to provide a comprehensive overview of ALI and ARDS, encompassing their definition, historical context, epidemiology, and common etiological factors, with a focus on recent advances in pathological mechanisms, signaling pathways, epigenetic regulation, and therapeutic interventions. Treatment approaches include lung-protective ventilation, fluid management, targeted pharmacotherapy, mesenchymal stromal cell therapy, and nanomaterial-based delivery systems. These developments offer valuable insights into the complex mechanisms of preclinical ALI and human ARDS and contribute to the advancement of clinical diagnosis and treatment.

## 1 Introduction

Acute lung injury (ALI) refers to experimental lung injury modeled in laboratory animals, while its corresponding severe clinical syndrome in humans is defined as acute respiratory distress syndrome (ARDS). First identified by Ashbaugh et al. in 1967, ARDS was described in 12 critically ill patients with acute tachypnea, refractory hypoxemia, reduced lung compliance, and diffuse bilateral pulmonary infiltrates on chest X-ray (1). In 1994, the American-European Consensus Conference established standardized diagnostic criteria for ALI/ARDS, defining ALI as acute bilateral pulmonary infiltrates with a PaO_2_/FiO_2_ratio ≤ 300 mmHg, excluding patients with clinical evidence of left atrial hypertension (2). The 2012 Berlin Definition further refined these criteria and stratified ARDS into mild, moderate, and severe subtypes based on hypoxemia severity. Notably, the Berlin criteria officially eliminated ALI as a clinical diagnostic category, reclassifying all previous mild ALI cases as mild ARDS (3). Currently, ALI is predominantly adopted for preclinical animal experimental models, as laboratory animals cannot fully recapitulate the complete clinical spectrum of human ARDS (4). In 2024, the Global ARDS Definition further expanded diagnostic applicability: it included non-intubated patients receiving high-flow nasal oxygen ≥ 20 L/min, allowed SpO_2_/FiO_2_< 315 (with SpO_2_≤ 97%) for oxygenation assessment, and provided simplified criteria suitable for resource-limited healthcare settings (5).

ARDS is a specific phenotypic subset of acute hypoxemic respiratory failure, and the two clinical conditions are not interchangeable. Diagnostic discrepancies between the Berlin and 2024 Global definitions significantly affect patient enrollment and the interpretation of clinical trial results. This review extensively cites preclinical data from rodent ALI models to elucidate core mechanistic pathways; nevertheless, inherent translational gaps persist between animal models and human ARDS, due to the inability of animal experiments to recapitulate the multifactorial heterogeneity, comorbidities, and dynamic clinical trajectories of human patients (4). Epidemiological data collected across ICUs in 50 countries demonstrated that ARDS accounted for 10.4% of ICU admissions and affected 23.4% of mechanically ventilated patients before the COVID-19 pandemic (6). The 2020–2022 COVID-19 outbreak triggered a sharp global rise in ARDS incidence and mortality (7, 8). Although the post-pandemic ARDS incidence has gradually stabilized, it remains markedly higher than pre-pandemic levels (9).

Clinically, ARDS is characterized by acute-onset severe dyspnea, tachypnea, oxygen-refractory hypoxemia, and bilateral pulmonary infiltrates on chest imaging (10). In the early exudative stage, patients develop progressive respiratory distress, decreased lung compliance, and non-cardiogenic pulmonary edema driven by alveolar accumulation of protein-rich exudate (10). The late proliferative and fibrotic stages are dominated by organizing pneumonia, interstitial fibrosis, and persistent pulmonary dysfunction (11). Pathologically, ALI and ARDS share core hallmarks of diffuse alveolar damage, including hyaline membrane formation, disruption of alveolar epithelial and endothelial barriers, and extensive neutrophilic infiltration (12). Beyond isolated lung injury, ARDS frequently induces extrapulmonary organ dysfunction. Approximately 75% of fatal ARDS cases are complicated by multiple organ dysfunction syndrome (MODS), involving the cardiovascular, renal, coagulatory, and hepatic systems (13, 14).

ALI and ARDS arise from multifactorial etiologies, which are conventionally categorized into direct (intrapulmonary) and indirect (extrapulmonary) insults (15). Direct triggers include severe pneumonia, gastric aspiration, lung contusion, inhalation injury, and drowning, whereas indirect causes primarily consist of sepsis, severe trauma, acute pancreatitis, massive blood transfusion, and extensive burns (16, 17). Among these risk factors, sepsis and pneumonia are the most prevalent, with sepsis alone accounting for 40-46% of all ARDS cases (18, 19). The COVID-19 pandemic caused by SARS-CoV-2 further elevated the global disease burden of ARDS, with 15-30% of hospitalized COVID-19 patients progressing to severe ARDS (20, 21). Despite continuous optimization of supportive care, lung-protective ventilation strategies, and standardized respiratory support protocols updated in the 2023 European Society of Intensive Care Medicine guidelines, ARDS mortality remains persistently high and positively correlated with disease severity (22, 23).

ARDS survivors commonly suffer from long-term adverse sequelae, including impaired quality of life, persistent neuromuscular weakness, cognitive dysfunction, depression, anxiety, and post-traumatic stress disorder (24, 25). Beyond individual patient harm, ALI and ARDS impose a substantial economic burden on global healthcare systems. A Spanish epidemiological and economic study on mechanically ventilated ARDS patients revealed that ARDS-related clinical and diagnostic costs have nearly quadrupled in recent years, with a peak per capita cost of 42812 euros and a stable subsequent range of 30000 to 40000 euros (9). Similar heavy economic burdens have been documented worldwide, confirming ARDS as a critical public health challenge that consumes massive healthcare resources (26).

The pathogenesis of ALI and ARDS are tightly regulated by multiple well-characterized signaling pathways (27). These signaling cascades independently or synergistically modulate inflammatory responses, oxidative stress, cell death and tissue repair processes in the injured lung (28). Accumulating clinical evidence points to marked biological heterogeneity among patients diagnosed with ARDS. Researchers use the term subphenotypes to describe patient subgroups identifiable through clinical and laboratory measurements. True endotypes, by contrast, require fully elucidated disease mechanisms that reliably predict treatment effects; for this reason most currently-described ARDS subgroups are considered candidate endotypes (29). Multiple stratification strategies are already in clinical use, including Berlin severity grading and the distinction between direct versus indirect lung injury. Among biomarker-derived subgroups reproduced across large cohorts, hyperinflammatory and hypoinflammatory subphenotypes remain the most thoroughly studied examples (30). Patients with the hyperinflammatory subphenotype frequently show elevated pro-inflammatory mediators, enhanced coagulation activation, higher multi-organ-dysfunction rates and worse outcomes, whereas hypoinflammatory cases display blunted inflammation and relatively lower mortality. These subgroups may respond differently to fluid management, ventilator settings and immunomodulatory therapies (30). Current ESICM guidelines highlight phenotypic and endotype-targeted stratified therapy as a core direction for future ARDS research, indicating that unified treatment regimens cannot adapt to heterogeneous ARDS patients. Accordingly, growing research focuses on exploring signaling modulators for ALI and ARDS intervention. This review systematically summarizes the latest research on ALI and ARDS etiologies and pathogenesis, with a key focus on dissecting pivotal signaling pathways driving disease progression. We also comprehensively discuss current clinical interventions, approved drugs, preclinical and clinical investigational therapeutic agents, and emerging targeted treatment technologies, aiming to provide updated insights for the precise diagnosis and treatment of ALI and ARDS.

## 2 Risk factors and symptoms

Over the past few decades, the etiology of ALI and ARDS has been extensively investigated, with substantial evidence demonstrating that both genetic susceptibility and environmental exposure contribute critically to disease pathogenesis (5, 10, 31). Multiple susceptibility genes, including *ACE*, *IL-6*, *SFTPB*, *VEGFA*, and *TNF*, have been confirmed to be closely associated with ALI and ARDS onset and prognosis (32–34). Among these, *ACE* is one of the most extensively studied candidates. It encodes a core enzyme of the renin-angiotensin system that modulates vascular permeability and pulmonary inflammatory responses (35). The insertion/deletion polymorphism of the *ACE* gene is correlated with mortality risk in Asian ARDS patients, though its association with disease susceptibility remains controversial across ethnic populations (36, 37). The *SFTPB* gene encodes surfactant protein B, an essential protein for maintaining alveolar stability, and represents another key susceptibility locus, particularly in patients with direct lung injury such as pneumonia (38, 39). Polymorphisms in inflammatory cytokine genes, including *IL-6*, *TNF*, and *VEGFA*, have also been repeatedly validated in independent cohorts to correlate with ALI and ARDS susceptibility and clinical outcomes (40–42).

Environmental factors are equally indispensable in modulating ALI and ARDS pathogenesis, with cigarette smoking recognized as a major modifiable risk factor (43). A meta-analysis of 26 observational studies encompassing 36995 participants confirmed that smoking significantly elevates ARDS risk (44). Chronic alcohol abuse also serves as an independent risk factor, increasing susceptibility to severe lung injury and contributing to higher mortality (45, 46). Furthermore, obesity, long-term air pollution exposure, and electronic cigarette use have emerged as novel modifiable environmental determinants of ALI and ARDS susceptibility (47–49). Collectively, the complex crosstalk among genetic polymorphisms, epigenetic modifications, and external environmental exposures ultimately determines individual vulnerability to ALI and ARDS following pulmonary or systemic insult.

Although ARDS predominantly manifests as a pulmonary disorder, it frequently progresses to systemic multi-organ dysfunction, involving the cardiovascular system, kidneys, liver, and central nervous system (6, 10). The syndrome is characterized by acute-onset respiratory deterioration, including severe dyspnea, tachypnea, and oxygen-refractory hypoxemia (50). Physical examination commonly presents with tachycardia, increased respiratory workload with accessory muscle activation, and bilateral diffuse rales on chest auscultation (10). These clinical manifestations typically emerge within 6 to 72 hours after the initiating insult and can rapidly progress to severe respiratory failure requiring intensive care unit admission and mechanical ventilation (51). Hypoxemia constitutes the central hallmark of ARDS. Notably, a unique clinical phenotype termed “silent hypoxemia” is frequently observed, particularly in COVID-19-associated ARDS patients, which is defined as profound hypoxemia accompanied by relatively preserved respiratory drive and minimal subjective dyspnea (3, 52, 53).

Persistent uncontrolled pulmonary inflammation drives progressive diffuse alveolar damage, hyaline membrane formation, and subsequent fibroproliferative remodeling, which may lead to irreversible pulmonary fibrosis in advanced stages (11, 54). The systemic inflammatory cascade triggered by ARDS further precipitates multiple organ dysfunction syndrome (MODS), among which cardiovascular, renal, and hematological dysfunction are the leading causes of mortality (55). Recent clinical evidence confirms that MODS remains the primary driver of death in ARDS patients, with elevated Sequential Organ Failure Assessment (SOFA) scores serving as an independent predictor of mortality in up to 60% of severe cases (56).

ARDS survivors suffer from long-term debilitating sequelae that severely impair their quality of life. A 2025 meta-analysis demonstrated that persistent cognitive impairment affects approximately 36% of survivors, while fibrotic pulmonary changes are detected in over 50% of patients one year after severe COVID-19-associated ARDS (57, 58). Additionally, long-term psychiatric complications, including anxiety, depression, and post-traumatic stress disorder (PTSD), are highly prevalent. These overlapping physical, cognitive, and psychological sequelae collectively constitute Post-Intensive Care Syndrome (PICS), which leads to sustained functional impairment and reduced health-related quality of life for years after hospital discharge (59).

## 3 The pathogenesis of ALI

ALI represents a progressive, interconnected pathological cascade rather than a collection of isolated molecular events. Its conceptual disease trajectory unfolds through a sequential series of pathological stages. Following initiating insults, injury occurs to alveolar epithelial and pulmonary endothelial cells, which subsequently causes breakdown of the alveolar-capillary barrier. Barrier disruption triggers activation of pulmonary innate immunity, giving rise to amplified neutrophil-dominated inflammation and immunothrombosis. These events are followed by oxidative stress, endoplasmic-reticulum stress and mitochondrial-driven metabolic stress (**Figure 1**). Secondary mechanical amplification of injury, namely ventilator-induced lung injury (VILI) and patient-self-inflicted lung injury (P-SILI), then ensues. This cascade terminates in one of two distinct biological outcomes: inflammatory resolution and tissue repair, or maladaptive pathological remodeling progressing to pulmonary fibrosis.

**Figure 1:**
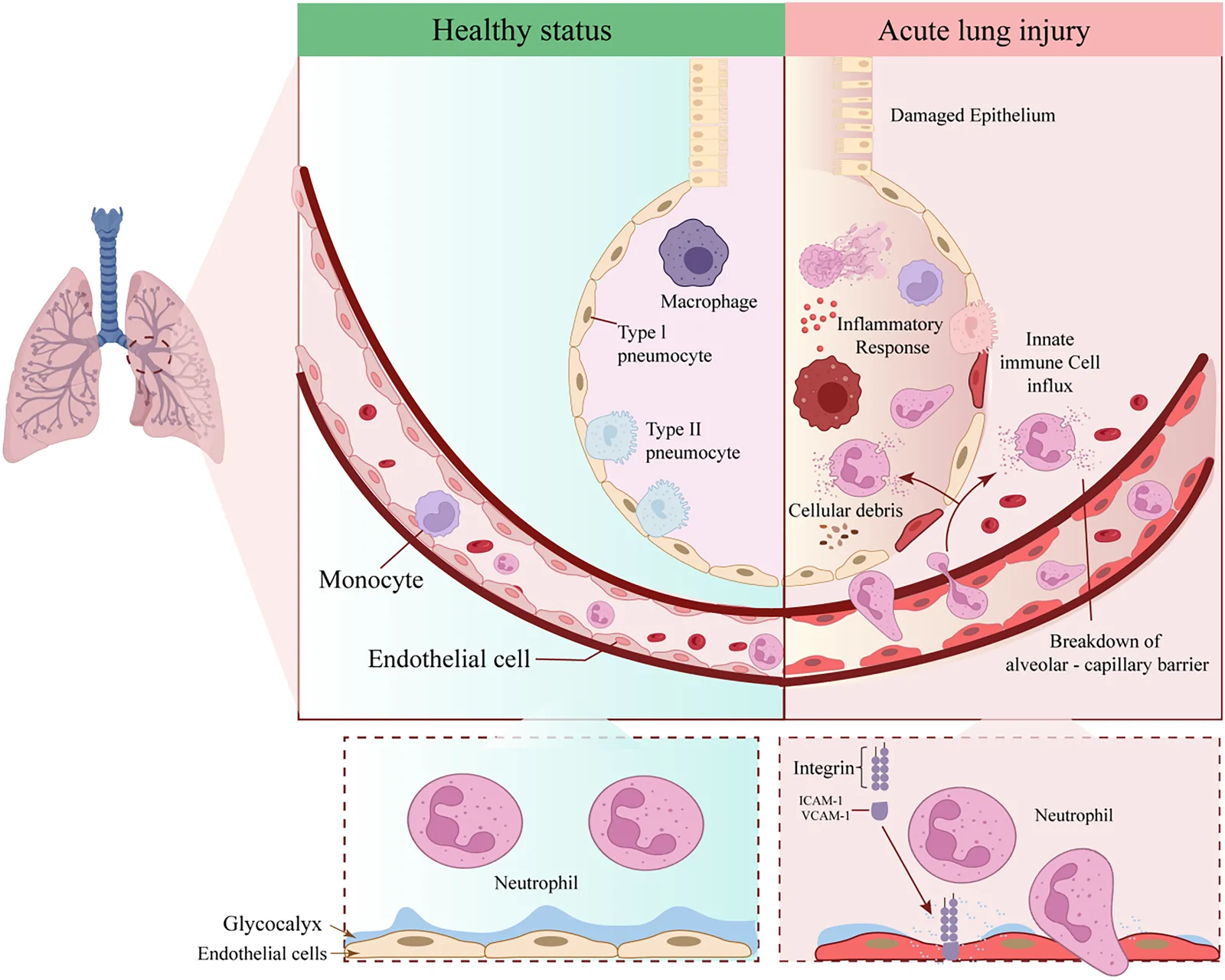
In healthy lung tissue, the respiratory epithelium remains fully intact, and the alveolar spaces are patrolled by resident alveolar macrophages. Alveolar type I and type II cells are responsible for regulating the alveolar milieu and maintaining the integrity of the alveolar-capillary barrier. During ALI and ARDS, innate immune cells such as neutrophils and monocytes migrate from the circulation into the interstitium and alveolar spaces. Damage to the epithelial and endothelial layers leads to disruption of the alveolar-capillary barrier, resulting in the accumulation of edematous fluid and debris within the alveolar lumen. The pro-inflammatory and tissue-destructive functions of alveolar macrophages, monocytes, and neutrophils collectively contribute to the disease pathology.

Multiple heterogeneous initiating triggers, ranging from bacterial or viral pneumonia, sepsis, and aspiration to trauma and SARS-CoV-2 infection, can launch this pathological sequence. A broad spectrum of molecular mediators, aberrant signaling cascades, organelle dysfunction, epigenetic reprogramming, and mechanical forces jointly fuel disease progression, driving barrier leakage, overwhelming inflammatory activation, cell death and parenchymal destruction. Mechanical stress from invasive ventilation or excessive patient-driven inspiratory effort may serve as a potent “second hit” that worsens existing lung damage. The ultimate fate of injured lung tissue hinges on the dynamic balance between pro-inflammatory injury programs and pro-resolution-repair pathways. The pulmonary endothelium acts as a central pathogenic hub in ALI and ARDS, integrating barrier failure, inflammatory signaling, leukocyte recruitment, and microvascular thrombotic events to drive impaired pulmonary gas-exchange function. Based on this integrated pathological cascade framework, the following sections elaborate the core molecular and cellular mechanisms driving ALI progression.

### 3.1 Alveolar-capillary barrier disruption

The earliest tangible pathological readout of ALI cascade is structural and functional failure of the alveolar-capillary barrier, driven by primary death and dysfunction of alveolar epithelial and pulmonary endothelial cells after initiating insults.

#### 3.1.1 Alveolar epithelial barrier disruption

The alveolar epithelial barrier consists of alveolar type I epithelial (AT1) cells, alveolar type II epithelial (AT2) cells, and intercellular tight junctions. In ALI, damage to both epithelial cell populations and dysfunctional repair mechanisms establish a vicious cycle of impaired injury-repair, ultimately leading to the collapse of alveolar barrier integrity.

AT1 cells are thin, plate-like cells that cover approximately 95% of the alveolar surface, forming a delicate and extensive barrier indispensable for efficient pulmonary gas exchange. AT1 cells express the receptor for advanced glycation end products (RAGE), which is predominantly distributed on the basolateral membrane. This receptor facilitates cell adhesion to collagen and promotes cell spreading, thereby maintaining alveolar epithelial barrier stability and normal gas exchange function (60). As cuboidal epithelial cells accounting for only 5% of the alveolar surface area, AT2 cells exert dual core functions: differentiating into AT1 cells to repair damaged epithelial barriers and synthesizing pulmonary surfactant to prevent alveolar collapse (61). The functional fate of AT2 cells presents prominent spatiotemporal heterogeneity across the inflammatory, repair, and fibrotic stages of ALI. In the inflammatory phase, AT2 cells serve as critical regulators of the lung immune microenvironment. They inhibit macrophage activation via the CD200-CD200R signaling axis (62, 63) and secrete complement C3 to alleviate excessive inflammatory responses (64). Specifically, a subset of AT2 cells with low surfactant protein C expression and high MHC-II expression can present antigens to pulmonary CD4^+^ tissue-resident memory T cells, thereby executing key immunomodulatory functions (65).

AT2 cells also sustain pulmonary immune homeostasis through metabolic reprogramming. Enhanced mitochondrial long-chain fatty acid β-oxidation in AT2 cells suppresses the secretion of neutrophil chemokine CXCL2, mitigating neutrophil-mediated pulmonary inflammation. Meanwhile, this metabolic pathway provides cellular energy and supports phospholipid synthesis for surfactant production (66). In addition, AT2 cells rely on mitochondrial calcium uniporter-mediated calcium buffering to promote surfactant secretion and relieve pulmonary edema (67). The exosomal pathway further mediates the long-distance immunomodulatory effects of AT2 cells. AT2 cell-derived exosomes enriched with miR-27b-3p target regulator of G protein signaling 1 in alveolar macrophages, modulating calcium signaling-dependent immune responses and maintaining alveolar immune homeostasis (68).

During the repair phase, the proliferation and differentiation of AT2 cells are precisely modulated by multiple signaling pathways. The Wnt/β-catenin pathway primarily drives AT2 cell proliferation (69), whereas the Notch pathway governs AT2-to-AT1 cell differentiation (70). A previous study from Morrisey’s team demonstrated that the transcription factor grainyhead-like 1 (GRHL1) exerts temporal-specific regulatory effects on AT2 cells: it inhibits AT2 cell proliferation at the early repair stage (day 10) but facilitates AT2 proliferation and AT1 differentiation at the late repair stage (day 14) (71). Furthermore, the activity of Hippo pathway effectors YAP/TAZ in AT2 cells is essential for functional alveolar epithelial repair and the inhibition of pulmonary fibrotic remodeling (72). Multiple factors, including inflammatory regulation, metabolic homeostasis, and telomerase stability, coordinately ensure ordered tissue repair (73).

Insufficient repair capacity traps AT2 cells in a transitional state characterized by high expression of Krt8, Krt18, and Cdkn1a, rendering them incapable of differentiating into mature AT1 cells (74). These transitional AT2 cells specifically express amphiregulin (75) and transforming growth factor-β (TGF-β) (76), which activate fibroblasts and drive the progression of pulmonary fibrosis.

#### 3.1.2 Pulmonary capillary barrier disruption

The pulmonary endothelium represents a central functional compartment of the alveolar-capillary unit, governing vascular permeability, fluid-solute homeostasis, leukocyte trafficking, coagulation balance, and pulmonary gas-exchange efficiency (77). Endothelial dysfunction constitutes a key early event in ALI, manifested by glycocalyx degradation, phenotypic endothelial activation, increased vascular permeability, dysregulated endothelial-leukocyte crosstalk, and pulmonary microvascular thrombosis, all of which collectively worsen gas-exchange impairment.

The structural and functional integrity of this barrier primarily relies on the endothelial glycocalyx (EG)—a dynamic network of membrane-bound proteoglycans, glycosaminoglycan chains, and adsorbed plasma proteins covering the luminal surface of endothelial cells. The EG serves not only as a physical and charge barrier regulating vascular permeability, microvascular tone, and leukocyte adhesion but also as a significant signaling platform. In ALI, reactive oxygen species (ROS) and pro-inflammatory cytokines drive glycocalyx shedding by activating sheddases such as heparanase, metalloproteinases, and hyaluronidases; glycocalyx degradation marks the onset of endothelial activation and represents a pivotal early step toward pathological changes including increased vascular permeability, vasomotor dysfunction, and enhanced leukocyte-endothelial interactions (78).

Upon initiating insults, pro-inflammatory mediators (e.g., LPS, TNF-α, ROS) trigger full phenotypic endothelial activation. Activated endothelial cells undergo profound cytoskeletal remodeling via the RhoA/ROCK signaling pathway. This remodeling encompasses actin stress-fiber formation (79, 80), microtubule disassembly (81), and phosphorylation of vimentin intermediate filaments (82), ultimately leading to endothelial cell retraction and intercellular gap formation. Concurrently, intercellular junction complexes are compromised: Src-dependent tyrosine phosphorylation of VE-cadherin induces its dissociation from the actin cytoskeleton (79, 83), accompanied by down-regulation of tight-junction proteins (e.g., claudin-5, ZO-1) (84, 85), and gap-junction dysfunction.

Endothelial activation further promotes the surface expression of adhesion molecules ICAM-1 and VCAM-1, enabling firm endothelial-leukocyte interactions that facilitate neutrophil recruitment and transmigration into lung parenchyma (86, 87). Meanwhile, activated endothelium shifts toward a pro-coagulant phenotype: loss of anticoagulant surface molecules together with exposure of sub-endothelial basement membrane initiates local coagulation cascades, giving rise to pulmonary microvascular thrombosis. Thrombin generation in turn reinforces endothelial barrier dysfunction via protease-activated-receptor feedback signaling, forming a vicious cycle linking endothelial injury, inflammation, and microvascular thrombosis (88, 89).

When injury reaches its peak, endothelial cells undergo distinct forms of regulated cell death including apoptosis (caspase-dependent non-lytic programmed cell death), necroptosis (caspase-independent regulated necrotic cell death), pyroptosis (inflammasome-driven inflammatory lytic cell death) and ferroptosis (iron-dependent lipid-peroxidation-mediated cell death), resulting in endothelial denudation. These are mechanistically distinct regulated cell-death modalities and are not physiologically interchangeable. Exposed vascular basement membrane further amplifies pro-coagulant and pro-inflammatory responses, aggravating pulmonary edema and hypoxaemia and directly impairing alveolar-capillary gas exchange (90, 91). Beyond paracellular gap formation, transcellular vesicle-mediated transcytosis substantially contributes to macromolecular leakage during early ALI. Pro-inflammatory mediators induce caveolin-1 phosphorylation and PV1 down-regulation through the TLR4/MyD88 axis, augmenting vesicular transport activity (92). This aberrant transcytosis cooperates with cytoskeletal rearrangement to produce early hyperpermeability, independent of complete junctional breakdown (93).

Taken together, pulmonary vascular leakage in ALI arises from the synergistic interplay of glycocalyx erosion, paracellular junction disassembly, enhanced transcellular transcytosis, and endothelial cell loss. Endothelial dysfunction is not merely a passive outcome of inflammation; it actively amplifies inflammatory cell recruitment, microvascular thrombosis and oedema formation, and ultimately mediates deterioration of pulmonary gas-exchange function.

### 3.2 Immune dysregulation

Following endothelial-driven alveolar-capillary barrier disruption, leakage of damage-associated molecular patterns (DAMPs) and pathogen-associated molecular patterns (PAMPs) into alveolar spaces triggers potent innate immune activation, the central amplification node of ALI progression. Endothelial activation also provides critical adhesive signals to facilitate the recruitment of circulating innate immune cells. This phase is characterized by massive neutrophil recruitment, neutrophil-driven tissue injury, immunothrombosis, and dysregulated macrophage polarization.

#### 3.2.1 Neutrophil dysfunction

In the pathological progression of ALI, aberrant activation, functional dysregulation, and impaired clearance of neutrophils constitute a central mechanism driving lung tissue injury and persistent inflammation. Stimulated pulmonary cells secrete ELR+ chemokines such as CXCL1 and CXCL8, which bind to CXCR2 (and partially CXCR1) on the neutrophil surface. This interaction activates the PI3K/Akt and MAPK pathways, promoting neutrophil adhesion, transendothelial migration, and directed chemotaxis into lung tissue (94, 95). Excessively activated neutrophils exacerbate inflammatory responses and lung tissue injury through multiple mechanisms. Firstly, they release serine proteases, matrix metalloproteinases, lactoferrin, highly reactive oxygen species, and cytotoxic molecules, which amplify intracellular inflammatory signaling cascades and subsequently drive the tissue-level inflammatory response (96, 97). Secondly, they generate neutrophil extracellular traps (NETs). While NETs can entrap pathogens during infection, their excessive and persistent release directly damages the alveolar epithelial-endothelial barrier, triggers immunothrombosis within pulmonary microvessels, and activates downstream pro-inflammatory signaling cascades. NET-derived DNA further fuels a self-amplifying “NET-cytokine” vicious cycle that sustains inflammatory storm and immune dysregulation (98). Notably, the DNA backbone of NETs can be internalized by cells like alveolar epithelial cells via endocytosis, activating the intracellular cyclic GMP-AMP synthase (cGAS)-STING pathway and subsequently inducing alveolar epithelial cell necroptosis, thereby aggravating lung injury and inflammation (99, 100). Moreover, neutrophils directly influence the inflammatory process by modulating macrophage phenotypes. Normally, apoptotic neutrophils expose phosphatidylserine on their surface, triggering efferocytosis by macrophages and promoting their polarization toward an anti-inflammatory M2 phenotype (101). Impaired apoptosis, however, favors NET formation, which in turn induces macrophage polarization toward a pro-inflammatory M1 phenotype (102). IL-10 plays a crucial negative feedback role in this process by accelerating neutrophil apoptosis (103).

Apart from neutrophil-mediated tissue injury, modern understanding of ALI and ARDS has moved past viewing this condition solely as an inflammatory lung disorder. Growing evidence indicates that harmful lung damage arises from close interactions between innate immune responses, endothelial injury, platelet activity and abnormal coagulation, a biological process termed immunothrombosis (31, 104).

Once the alveolar-capillary barrier fails, inflammatory mediators including TNF-α, IL-1β, IL-6, DAMPs and PAMPs first alter endothelial cell behavior, while simultaneously recruiting neutrophils and activating circulating platelets. In response to inflammatory stimuli, the endothelial glycocalyx degrades. Endothelial cells lose surface anticoagulant proteins such as thrombomodulin and endothelial protein C receptor, and raise expression of adhesion molecules (ICAM-1, VCAM-1) as well as tissue factor, shifting toward a pro-adhesive and clot-promoting phenotype (78, 88). These endothelial changes in turn serve two major pathological purposes: they enable neutrophils to adhere and migrate across vessel walls into lung tissue, and they provide surface conditions that drive platelet accumulation and activation within the pulmonary microvasculature.

Activated platelets further amplify neutrophil-driven injury. Binding between platelet P-selectin and neutrophil PSGL-1 promotes NET release; these NET structures consist of chromatin DNA, histones, neutrophil elastase and myeloperoxidase (105, 106). NETs inflict lung injury through two distinct routes. Their histones and proteases directly damage alveolar epithelial and endothelial layers and further weaken barrier integrity. Meanwhile, the negatively-charged DNA strands of NETs act as a physical scaffold to trap circulating platelets, von-Willebrand factor and clot-related proteins, which then supports the formation of small microthrombi inside lung capillaries (107).

As microthrombi build up in pulmonary microvessels, they disrupt gas exchange by worsening ventilation-perfusion mismatch, increasing dead-space ventilation and exacerbating hypoxemia. Microthrombi also trigger additional release of DAMPs, which stimulates further local inflammation. Importantly, this damage is not one-sided: thrombin generated during local clot formation acts back on endothelial cells via protease-activated receptors (PARs). This increases endothelial permeability and induces chemoattractant production, which draws more neutrophils and sustains ongoing NET release (89). Activated platelets compound this effect by secreting large quantities of inflammatory mediators alongside plasminogen-activator inhibitor-1 (PAI-1). PAI-1 suppresses local clot breakdown and keeps these microthrombi persistent within lung microcirculation (108).

Notably from a clinical perspective, patients with the hyperinflammatory ARDS subphenotype show higher circulating levels of biomarkers for NET release, platelet activation and coagulation activity. Greater immunothrombosis burden correlates with worse clinical outcomes and higher risk of multiple-organ injury (31). It is worth noting that controlled immunothrombosis represents a normal host-defence mechanism that helps contain invading pathogens during infection. However, when reciprocal activation among NETs, platelets and endothelial cells cannot be resolved, this protective host response turns destructive and continuously impairs the alveolar-capillary barrier in ALI and ARDS. For these reasons, exploring therapeutic strategies targeting key links within this immune-endothelial-clotting interplay—such as limiting NET release, interfering with platelet-neutrophil contacts or lowering the clot-promoting properties of injured endothelium—is a promising direction for future ALI research.

#### 3.2.2 Imbalance in macrophage polarization

Pulmonary macrophages primarily consist of alveolar macrophages (AMs) and interstitial macrophages (IMs). AMs, predominantly tissue-resident and exhibiting an M2-like phenotype under homeostatic conditions (109), constitute the first line of defense in the alveolar space (110). They maintain pulmonary immune homeostasis by secreting anti-inflammatory factors (e.g., IL-10, TGF-β) and clearing surfactant. IMs reside in the lung parenchyma between the microvascular endothelium and the alveolar epithelium. They participate in tissue remodeling, maintain lung homeostasis and antigen presentation, and influence dendritic cell function to prevent airway allergy (111). However, under pathological stimuli such as infection, trauma, or sepsis, the polarization balance of pulmonary macrophages is profoundly disrupted. This imbalance exhibits “temporal-spatial dependent” characteristics and persists throughout the exudative, reparative, and fibrotic phases of ALI (112), emerging as a central driver of lung tissue injury and disordered repair.

During the acute exudative phase of ALI, pathogenic signals such as LPS and IFN-γ induce rapid polarization of resident AMs toward an M1 phenotype by activating signaling pathways like TLR4/NF-κB and JAK/STAT1 (113). Concurrently, peripheral blood monocytes are recruited into the alveolar space and differentiate into pro-inflammatory M1 macrophages (114, 115). M1 macrophages exhibit high expression of inducible nitric oxide synthase (iNOS) and cyclooxygenase-2 (COX-2), and release substantial amounts of inflammatory mediators such as TNF-α, IL-1β, IL-6, and ROS (116, 117). These mediators exacerbate inflammatory cell infiltration into the alveolar space by activating neutrophil chemotaxis and promoting their trans-endothelial and -epithelial migration (118). Furthermore, excessive iNOS-mediated NO production and ROS burst directly damage the alveolar epithelial and vascular endothelial barriers, leading to increased alveolar-capillary permeability, alveolar edema, and hemorrhage, thereby establishing a vicious cycle of “inflammatory storm” and tissue injury (119). As the initiating insult subsides and the process enters the reparative phase, a critical shift in the microenvironment occurs. Factors such as TNFα-stimulated gene-6 and MCP-induced protein 1 promote macrophage polarization toward an M2 phenotype (118). M2 macrophages facilitate resolution and repair through multiple mechanisms. On one hand, they can induce the generation of CD4+CD25+Foxp3+ regulatory T cells (Tregs) and help maintain their un-impaired immunosuppressive function, thereby modulating the immune response (120). On the other hand, they upregulate the expression of IL-10, fibronectin 1, TGF-β-induced matrix-associated protein BIG-H3, and insulin-like growth factor 1, which helps limit pro-inflammatory cytokine levels and promote tissue repair (121). Moreover, exosomal PGE2 derived from M2 macrophages can inhibit neutrophil migration and NET formation (122). Additionally, the transient receptor potential vanilloid 4 channel senses changes in extracellular matrix (ECM) stiffness following lung injury and synergizes with LPS-induced signaling to accelerate the phagocytic rate of apoptotic neutrophils by M2 macrophages, thereby terminating inflammatory signals (123). However, insufficient or delayed M2 polarization during the repair phase can lead to protracted inflammation and increased risk of secondary infection. Conversely, excessive or persistent M2 polarization can drive the progression of ALI toward a fibrotic phase. Chronically activated M2 macrophages secrete tissue inhibitors of metalloproteinases that inhibit the activity of matrix metalloproteinases (MMPs), leading to an imbalance in ECM degradation (121). Furthermore, they secrete IL-4, IL-13, and TGF-β, which can activate lung fibroblasts to transform into myofibroblasts, promoting collagen deposition and destruction of alveolar architecture (124).

However, the imbalance in pulmonary macrophage polarization is not a simple “M1/M2 dichotomy” but involves “phenotypic plasticity” and a “functional paradox”, which are contingent on the specific microenvironment (125). For instance, during the exudative phase of ALI, a subset of M1 macrophages may protect the alveolar epithelial barrier by expressing amphiregulin (126). Conversely, in the fibrotic phase, IL-4-induced M2 macrophages can inhibit fibrosis through the expression of Arg-1 and resistin-like molecule α (127). These observations indicate a dissociation between polarization phenotypes and their functions in specific contexts. Thus, macrophages paradoxically participate in the processes of inflammation, tissue repair, and fibrosis during ALI.

#### 3.2.3 Other key immune subsets, complement and immunothrombosis

Beyond neutrophils and macrophage populations, multiple additional immune cell subsets contribute to human ALI and ARDS immunopathology, which remain incompletely characterized in pre-clinical rodent models (128). Innate-like γδ T17 cells, tissue-resident memory T cells, cytotoxic CD8^+^ T cells, natural killer cells and dendritic cells coordinate early local inflammatory responses, antigen presentation and subsequent tissue repair; γδ T17 cells in particular can rapidly secrete IL-17 to amplify pulmonary inflammation upon tissue injury (129). B-cell-derived pathogenic antibodies may also aggravate epithelial-endothelial injury in certain ARDS contexts, though their clinical relevance varies across etiologies (130). Circulating monocytes exhibit substantial functional heterogeneity: classical, intermediate and non-classical monocyte subsets display divergent chemokine responsiveness, cytokine production and tissue-recruiting properties during human ARDS, and monocyte-derived macrophage states cannot be fully simplified to the binary M1/M2 classification (131).

The complement cascade constitutes a major upstream inflammatory effector axis in human ARDS. Anaphylatoxin C5a drives neutrophil chemotaxis, NET formation, endothelial activation and immunothrombosis; complement membrane-attack complex C5b-9 further worsens alveolar-capillary barrier disruption and pulmonary microvascular injury (132). This mechanistic background underpins the clinical development of anti-C5a therapeutic vilobelimab for COVID-19-associated ARDS, yet complement-targeted strategies for non-COVID-19 ARDS still lack robust prospective human validation (133). Platelet-neutrophil and platelet-monocyte crosstalk further fuels immunothrombosis, linking innate immune activation to microvascular fibrin deposition, ventilation-perfusion mismatch and multi-organ injury in ARDS patients (134).

Recent human single-cell and spatial transcriptomic studies have uncovered high-resolution cellular states, inter-cellular communication and spatial organization of lung immune compartments in ARDS patients, revealing patient-specific immune endotypes that bulk transcriptomic approaches cannot resolve. Nevertheless, these omics findings remain largely confined to research settings, and translation to routine clinical biomarkers or patient-tailored therapies is still in early stages (135).

### 3.3 Endoplasmic reticulum stress and mitochondrial dysfunction

Concurrent with immune-driven inflammation, injured parenchymal and immune cells develop profound endoplasmic reticulum stress and mitochondrial dysfunction, constituting major sources of oxidative and metabolic stress within the ALI pathological cascade.

#### 3.3.1 Endoplasmic reticulum stress

In the pathological progression of ALI, endoplasmic reticulum (ER) stress emerges as a central mechanism driving inflammatory amplification and tissue damage. It orchestrates the functions of key cells, including alveolar epithelial cells and macrophages, by activating the three core pathways of the unfolded protein response (UPR): PKR-like endoplasmic reticulum kinase (PERK), inositol-requiring enzyme 1α (IRE1α), and activating transcription factor 6 (ATF6) (136). Specifically, upon disruption of ER proteostasis by factors such as ROS, LPS, and inflammatory stimuli, the PERK pathway is activated and phosphorylates eukaryotic translation initiation factor 2α. This leads to the selective translation of activating transcription factor 4 and C/EBP homologous protein (CHOP) (137). CHOP subsequently mediates alveolar epithelial cell apoptosis by downregulating the anti-apoptotic protein Bcl-2 and upregulating pro-apoptotic proteins like BIM/BAX, thereby compromising pulmonary barrier integrity (138). Activation of the IRE1α pathway results in the splicing of X-Box-binding protein-1 (XBP1) mRNA to generate the transcriptionally active mature protein XBP1s, which regulates the expression of pro-inflammatory and fibrosis-related genes (139). Furthermore, IRE1α forms a complex with TNF receptor-associated factor 2, activating the ER stress-specific apoptotic executioner caspase-12 (136) as well as the JNK/ASK1 pathway. This promotes the activation of pro-apoptotic proteins such as BIM and BAX, exacerbating cell death (140, 141). Following its cleavage in the Golgi apparatus, ATF6 translocates to the nucleus and binds to XBP1. This upregulates chaperones such as BiP, calreticulin (CRT), and protein disulfide isomerase to restore protein folding (142). However, aberrant activation of ATF6 can enhance apoptotic signaling by upregulating CHOP (143). Moreover, ER-stress-driven inflammatory signaling exacerbates lung tissue inflammation and microvascular leakage by modulating macrophage polarization (promoting pro-inflammatory cytokine release in M1 macrophages and inducing fibrotic mediator secretion in M2 macrophages) and activating the NLRP3 inflammasome to propagate the tissue-level inflammatory response (144). These mechanisms collectively propel the pathological advancement of ALI.

#### 3.3.2 Mitochondrial dysfunction

Mitochondria, serving as the cellular “powerhouses”, primarily drive adenosine triphosphate (ATP) synthesis through the oxidative phosphorylation process mediated by the electron transport chain (ETC), thereby maintaining cellular energy homeostasis. Beyond energy production, mitochondria play a crucial role in preserving cellular structural integrity, regulating alveolar surfactant secretion, and facilitating the repair of alveolar epithelial and endothelial barriers. Consequently, mitochondrial dysfunction is recognized as a central pathogenic mechanism in the development of ALI (145). In ALI, injurious stimuli such as LPS induce mitochondrial fragmentation. This occurs through the phosphorylation and activation of the fission protein Drp1 at Ser^616^ and the downregulation of fusion proteins Mfn1, Mfn2, and OPA-1 (146, 147). Fragmented mitochondria exhibit impaired oxidative phosphorylation, leading to insufficient ATP generation. This energy deficit compromises fundamental cellular functions, including surfactant secretion by alveolar epithelial cells and the stability of tight junctions in endothelial cells. Furthermore, fragmented mitochondria leak excessive amounts of mitochondrial reactive oxygen species (mtROS) from the compromised ETC (148, 149). Additionally, inhibition of coenzyme Q, a core component of the ETC, further promotes cytochrome c release and activates caspase-3/9, thereby enhancing inflammasome activity and the secretion of pro-inflammatory factors (150). mtROS exerts multiple detrimental effects during ALI pathogenesis. It directly induces lipid peroxidation in lung cell membranes, damaging alveolar-capillary barrier permeability. Simultaneously, mtROS activates the NF-κB signaling pathway, promoting the transcriptional expression of pro-inflammatory cytokines such as TNF-α and IL-6, and enhances the immunogenicity of mitochondrial DNA (mtDNA). Released mtDNA from damaged mitochondria enters the cytosol, where it amplifies the inflammatory cascade by activating pathways including the cGAS-STING pathway, TLR9 signaling, and inflammasomes (151). The expression level of mtDNA can serve as a biomarker for assessing disease severity (152). Furthermore, PINK1/Parkin-mediated mitophagy in ALI exhibits bidirectional dysregulation. Moderate mitophagy can be protective by clearing damaged mitochondria. However, excessive activation leads to the loss of functional mitochondria. Conversely, activation of the MKK3/p38 signaling pathway inhibits Parkin activity, impairing the clearance capacity of mitophagy and resulting in the accumulation of dysfunctional mitochondria (153, 154). Concurrently, overexpression of uncoupling protein 2 exacerbates mitochondrial uncoupling, reducing membrane potential and ATP synthesis, which further aggravates oxidative stress and inflammatory responses. These processes collectively disrupt pulmonary barrier integrity (155). Traditionally, it was believed that cells could only repair their own mitochondria through autophagy or biogenesis. However, recent studies indicate that mitochondria can be actively transferred between cells, forming an “intercellular rescue” network, and mitochondrial transplantation offers a novel therapeutic perspective for ALI (156).

### 3.4 Mechanical amplification of injury: VILI and P-SILI

After primary lung injury occurs, mechanical forces act as a potent “second-hit” amplifier in the ALI pathological cascade. Mechanical ventilation is not merely a clinical supportive technique but also serves as a biological modifier of pulmonary inflammation and cellular damage (157). Mechanical injury originates from VILI due to inappropriate ventilator settings and P-SILI driven by excessive spontaneous inspiratory effort, which can even occur under non-invasive respiratory support (158).

Given the pronounced spatial heterogeneity of injured lungs in ALI, delivered tidal volume is unevenly distributed across lung compartments. Well-aerated regions develop regional overdistension, while dependent consolidated alveoli undergo repetitive opening-and-closing cycles that generate injurious shear forces known as atelectrauma (159). These pathological events can be quantified using fundamental mechanobiology parameters: stress (force per unit area, approximated *in vivo* by transpulmonary pressure; note that transpulmonary pressure reflects forces acting directly upon lung tissue and is physiologically distinct from airway pressure and plateau pressure measured at the airway opening) and strain (the magnitude of tissue deformation) (160). Cumulative energy transferred to lung tissue per unit time, defined as mechanical power (cumulative energy transferred to lung tissue per unit time, units: J/min), has been demonstrated to independently predict worse patient outcomes (161). Excessive tissue deformation triggers mechanotransduction, converting physical mechanical stimuli into intracellular biochemical signals. Mechanosensitive ion channels and cytoskeletal sensors activate inflammatory cascades including NF-κB, MAPK and NLRP3 inflammasome, illustrating the interaction of mechanical stress with inflammatory signaling pathways (162). This so-called biotrauma promotes DAMP release, neutrophil recruitment, cell-junction disruption and microvascular thrombosis, establishing self-amplifying feed-forward injury loops.

P-SILI stems from forceful patient-generated inspiratory effort, producing harmful transpulmonary pressure swings, pendelluft, and heterogeneous stress-strain distribution even with nominally safe ventilator parameter settings (158). This mechanical amplification loop shapes disease trajectory: adequate lung-protective ventilation constrains stress-strain, lowers mechanical power, and mitigates excessive spontaneous inspiratory effort to limit both VILI and P-SILI and favor inflammatory resolution. In contrast, injurious mechanical stress perpetuates tissue injury and drives fibroproliferative remodeling.

### 3.5 Metabolic reprogramming

Oxidative stress, inflammatory signaling and mechanical strain collectively reshape cellular metabolism of immune and parenchymal cells, driving widespread metabolic reprogramming in the ALI cascade.

In ALI, metabolic reprogramming of macrophages plays a crucial role in both the initiation and resolution of inflammatory responses by regulating their polarization states. LPS stimulation triggers a shift in the metabolic phenotype of macrophages. This involves a transition from a resting state reliant on mitochondrial oxidative phosphorylation (OXPHOS) and fatty acid oxidation to a metabolic mode predominated by aerobic glycolysis (163). This process is characterized by the upregulation of key glycolytic molecules such as glucose transporter 1, pyruvate kinase M2, and 6-phosphofructo-2-kinase/fructose-2,6-bisphosphatase. This enhances glucose uptake and lactate production, providing the necessary bioenergy to fuel the rapid proliferation and robust cytokine production characteristic of M1 macrophage polarization (164, 165). Growing evidence indicates that glycolytic reprogramming— marked by enhanced glycolysis and dampened OXPHOS—is intricately linked to the activation of the NLRP3 inflammasome in macrophages (166). On one hand, accelerated glucose uptake and lactate accumulation provide both energy and metabolic substrates for NLRP3 inflammasome activation. Furthermore, the accumulation of the glycolytic intermediate succinate can stabilize HIF-1α, further promoting NLRP3 inflammasome activation (167). On the other hand, signaling pathways such as TLR4/NF-κB, AMPK, mTOR, and cGAS-STING can indirectly regulate NLRP3 inflammasome activation by modulating the expression of key glycolytic molecules (168). Beyond glycolytic reprogramming, LPS can also induce systemic lipid metabolism dysregulation. Specifically, it promotes the conversion of linoleic acid to arachidonic acid through catalysis by Δ6-desaturase and elongase. Arachidonic acid is subsequently metabolized via the cyclooxygenase (COX-1/COX-2) pathway to prostaglandin G2 and H2 (PGH2). PGH2 is ultimately converted to prostaglandin I2 by prostacyclin synthase and also undergoes non-enzymatic hydrolysis to form the stable metabolite 6-keto-prostaglandin F1α. This represents a novel pathogenic cascade that contributes to neutrophil infiltration, cytokine storm, and oxidative stress responses (169).

### 3.6 Cellular senescence, aging-associated susceptibility and SASP

Advanced age is a well-recognized non-modifiable risk factor for poor clinical outcomes in ALI and ARDS. Aging increases host susceptibility to pulmonary insults and disturbs the homeostatic control of inflammatory and tissue-repair programs (170). Cellular senescence is an important biological modifier that determines injury severity and tissue regenerative capacity during ALI. It refers to a stable and mostly irreversible state of cell-cycle arrest triggered by various stressors including DNA damage, mitochondrial dysfunction, endoplasmic reticulum stress, and pathogen-derived insults, all of which frequently occur in the course of ALI (171). Senescent alveolar epithelial cells (mainly AT2 progenitor cells), pulmonary endothelial cells, and immune cells accumulate within aged lung tissue. Moreover, acute lung injury itself can further induce new-onset cellular senescence. Senescent cells exert biological effects mainly through the senescence-associated secretory phenotype (SASP), which releases large quantities of pro-inflammatory cytokines, chemokines, and pro-fibrotic mediators via autocrine and paracrine pathways (172).

SASP-derived mediators amplify local pulmonary inflammation, disrupt the integrity of the alveolar-capillary barrier, and trigger “bystander senescence”. This process spreads senescence signals to adjacent normal cells and establishes a self-reinforcing pathological positive-feedback loop. Senescent AT2 progenitor cells display defective proliferation and reduced capacity to differentiate into AT1 cells. These defects delay alveolar epithelial repair and increase the likelihood of progression toward post-injury pulmonary fibrosis (173). Functionally, SASP promotes macrophage polarization toward the pro-inflammatory M1 phenotype and contributes to excessive neutrophil activation, which connects closely to the immune dysregulation described in Section 3.2. As mentioned in Section 3.3, mitochondrial dysfunction and endoplasmic reticulum stress act as upstream triggers for cellular senescence. In turn, mediators released by SASP can further worsen damage to these organelles, creating mutual regulatory interactions among different pathological processes of ALI (172).

Cellular senescence and SASP exert dual effects that depend on disease context. Transient senescence responses in the early injury phase can limit the expansion of irreversibly damaged cells. However, persistent accumulation of senescent cells and sustained SASP production mostly drive tissue damage and hamper the resolution of inflammation, contributing to long-term pulmonary complications such as post-ARDS pulmonary fibrosis, which is particularly prominent in aged populations (174). Therapies targeting senescent cell burden, including senolytics and senomorphic agents, have shown protective effects in pre-clinical aged ALI animal models by restraining disordered inflammatory responses and improving lung repair. Nevertheless, evidence regarding optimal treatment time windows and clinical translation remains insufficient.

## 4 Signaling pathways in the pathogenesis of ALI

The disease progression of ALI involves multiple signal transduction pathways. The major pathways are illustrated in **Figure 2**, where dysregulated signaling often serves as critical therapeutic targets for drug development.

**Figure 2:**
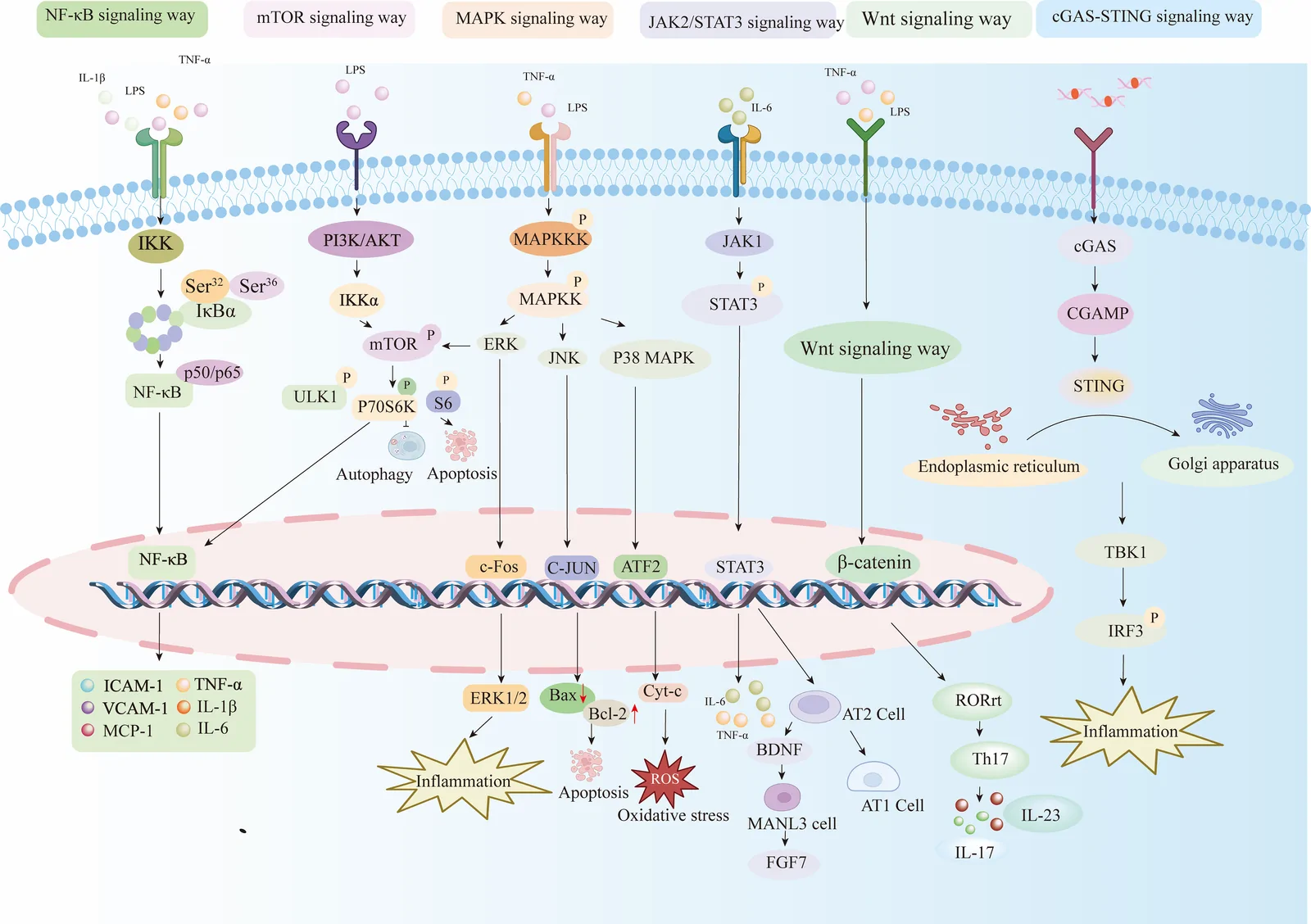
Major signaling pathways associated with ALI. The NF-κB signaling pathway, MAPK signaling pathway, JAK2/STAT3 signaling pathway, mTOR signaling pathway, Wnt-β-catenin signaling pathway, and cGAS-STING signaling pathway are the main signaling pathways involved in the process of ALI.

### 4.1 The NF-κB signaling pathway

Under physiological conditions, NF-κB dimers (predominantly the p50-RelA/p65 heterodimer) are sequestered in the cytoplasm of endothelial cells in an inactive state through binding to inhibitory proteins, IκBs. When lung tissue is stimulated by conditions such as pneumonia, sepsis, or trauma, mediators including TNF-α, IL-1β, LPS, or thrombin trigger upstream signaling cascades via receptors such as TNFR, PAR-1, and TLR4. This leads to the activation of the IκB kinase (IKK) complex (175, 176). Activated IKK phosphorylates the inhibitor κBα (IκBα) at Ser³² and Ser^36^, targeting it for ubiquitination and subsequent degradation via the proteasome pathway. The released NF-κB dimer, now exposing its nuclear localization signals, translocates to the nucleus. There, it binds to κB sites in the promoter regions of target genes (177), driving the transcriptional expression of adhesion molecules such as E-selectin, ICAM-1, and VCAM-1; chemokines including IL-8 and monocyte chemoattractant protein-1; and pro-inflammatory cytokines like TNF-α, IL-1β, and IL-6 (178). These effector molecules further promote EC activation, shifting their phenotype from an anti-adhesive, anticoagulant steady state to a pro-adhesive state. Consequently, activated ECs can capture circulating PMNs, decelerating them to a rolling state and ultimately leading to their firm adhesion. Subsequently, the adhered PMNs undergo spreading, crawling, and localization to transmigrate across the endothelium to the inflammatory site. This process disrupts EC junctional structures, increases vascular permeability, and results in the formation of protein-rich pulmonary edema (179). Furthermore, NF-κB activation downregulates anticoagulant proteins on the EC surface, such as thrombomodulin and the endothelial protein C receptor, thereby enhancing local coagulation activity and creating a vicious “inflammation-coagulation” cycle that exacerbates lung tissue injury (178, 180). It is noteworthy that NF-κB activation occurs via two main pathways: the canonical and non-canonical. In ALI, the canonical pathway, primarily responsible for regulating inflammation and cell survival, predominates. In contrast, the non-canonical pathway relies on NF-κB-inducing kinase to activate IKKα homodimers. IKKα then phosphorylates the NF-κB protein p100, stimulating its processing into p52. This pathway is mainly involved in gene expression related to B-cell maturation and lymphoid organogenesis and plays a weaker direct role in regulating acute inflammation (179). Simultaneously, NF-κB exhibits a “double-edged sword” characteristic during the course of ALI. During the resolution phase, NF-κB contributes to tissue repair and homeostasis restoration by inducing the formation of p50/p50 homodimers and the expression of anti-apoptotic and repair-related genes. Inhibiting NF-κB during this phase can aggravate endothelial barrier injury and delay tissue repair (181). This dual nature presents a significant challenge for the precise therapeutic targeting of NF-κB in ALI.

### 4.2 The MAPK signaling pathway

The Mitogen-Activated Protein Kinase (MAPK) signaling pathway, comprising the ERK, JNK, and p38 MAPK subfamilies (182), is a classic pathway mediating uncontrolled inflammation and tissue damage in ALI. LPS can initiate the cascade activation of the MAPK pathway by engaging receptors such as CD14 on the surface of immune cells like alveolar macrophages. This is manifested by significant elevations in the levels of phosphorylated ERK, JNK, and p38-MAPK (183). Phosphorylated and activated MAPK family members amplify pulmonary inflammation through several core mechanisms. They can directly translocate to the nucleus to regulate the gene transcription and protein secretion of pro-inflammatory factors such as TNF-α, IL-6, and IL-1β. Alternatively, they can cross-talk with the AKT/NF-κB signaling pathway, further enhancing IκBα phosphorylation/degradation and p65 nuclear translocation, thereby creating a synergistic effect that amplifies inflammation (184, 185). Furthermore, MAPK signaling is involved in modulating the activation of nuclear factor erythroid 2-related factor 2 (Nrf2); however, its excessive activation can exacerbate lung tissue inflammation and oxidative stress injury (186). Additionally, sustained activation of the MAPK pathway promotes increased activity of myeloperoxidase, aggravating oxidative stress damage and further propelling the pathological progression of ALI (183).

### 4.3 The JAK2/STAT3 signaling pathway

The JAK2 (Janus-activated Kinase2)-STAT3 (Signal Transduction and Activator of Transcription3) signaling pathway is activated by cytokine stimulation and transduces biological signals to target cells, regulating downstream genes involved in cell proliferation, inflammation, and fibrosis (187). In ALI and ARDS, the JAK2/STAT3 pathway participates in disease pathogenesis and progression through a “dual regulatory” mode. IL-6, a core pro-inflammatory mediator within the cytokine storm, activates JAK family kinases (predominantly JAK2) upon binding to its cell membrane receptor, leading to the phosphorylation of downstream STAT3 (188). Phosphorylated STAT3 translocates to the nucleus, binds to promoters of pro-inflammatory target genes, and drives the sustained release of inflammatory factors such as TNF-α and IL-6 (189). Concurrently, it promotes macrophage polarization toward the M1 phenotype, resulting in uncontrolled inflammation that further exacerbates ALI (190). Conversely, during the repair phase, STAT3 plays a crucial role in promoting alveolar epithelial regeneration. Specifically, STAT3 increases the expression of the growth factor brain-derived neurotrophic factor (BDNF) in transdifferentiating AT2 cells. BDNF acts as a key paracrine signaling molecule, binding to the TrkB receptor on mesenchymal alveolar niche cells. This activates downstream signaling and promotes the secretion of fibroblast growth factor 7 (FGF7). FGF7, in turn, acts back on AT2 cells, enhancing their proliferative capacity and inducing their transdifferentiation into AT1 cells, thereby facilitating alveolar epithelial regeneration and repair (191). Furthermore, TrkB agonists (e.g., 7,8-dihydroxyflavone) can enhance this signaling axis, improving the proliferation efficiency of AT2 cells, reducing the formation of abnormal repair-associated keratin 5-positive (Krt5^+^) cell clusters, and ameliorating lung histopathology and gas exchange function (191). Thus, STAT3 activation in AT2 cells plays a central role in repair processes.

### 4.4 The mTOR signaling pathway

The mammalian target of rapamycin (mTOR) is an atypical serine/threonine protein kinase involved in various cellular processes, including metabolism, proliferation, and autophagy (192). It exists as two distinct complexes, mTORC1 and mTORC2. mTORC1 stimulates mRNA translation, protein synthesis, and inhibits autophagy, whereas mTORC2 promotes cell survival, cytoskeletal reorganization, and metabolic regulation (193).

Inducers of ALI can modulate mTOR activity through upstream signaling, thereby influencing lung tissue injury and repair. For instance, stimuli can activate mTORC1 via pathways such as PI3K/AKT and MAPK/ERK, or inhibit mTOR activity through AMPK, REDD1, and p53 pathways. This differential regulation subsequently inhibits or promotes autophagy, respectively. Studies demonstrate that LPS stimulation activates the PI3K/AKT/mTOR signaling axis in pulmonary epithelial cells, leading to the phosphorylation of mTOR and its downstream substrates ULK1, p70S6K, and S6. This activation exerts dual detrimental effects: it inhibits autophagic initiation and flux completion, resulting in LC3-II accumulation and impaired P62 degradation, thereby hindering the clearance of damaged organelles and inflammatory mediators. Simultaneously, it promotes the activation of the TLR4/NF-κB pathway, exacerbating the release of pro-inflammatory factors such as IL-1β and TNF-α, and induces the cleavage and activation of caspase-3/7/9 and PARP, enhancing epithelial cell apoptosis. These combined effects ultimately disrupt alveolar structural integrity, increase pulmonary vascular permeability, and promote inflammatory cell infiltration (194). Conversely, activated p53 can induce autophagy by directly downregulating the phosphorylation levels of mTOR and its downstream substrates (195). Furthermore, exogenous stimuli can activate mTORC1, enhancing protein synthesis. This leads to the accumulation of unfolded/misfolded proteins within the endoplasmic reticulum, thereby activating the unfolded protein response (196). In summary, excessive activation of the mTOR pathway serves as a key regulatory node connecting endoplasmic reticulum stress, autophagy, apoptosis, and endothelial barrier dysfunction in ALI. Targeted inhibition of this pathway may confer protective effects against ALI.

### 4.5 Wnt-β-catenin signaling pathway

The Wnt-β-catenin signaling pathway is crucial for cell growth and proliferation and plays a complex “double-edged sword” role in ALI, with its specific effects contingent on the phase of injury. Under pulmonary homeostasis, Wnts secreted by neighboring PDGFRα+ fibroblasts maintain ATII cell identity and phenotypic stability, as knockout of β-catenin leads to their differentiation into ATI cells (197). During the acute injury phase, activated Wnt-β-catenin signaling can promote inflammation. It does so by recruiting the histone acetyltransferase p300 to modulate the transcriptional activity of retinoic acid-related orphan receptor γt, thereby enhancing the proliferation and activation of Th17 cells and increasing the secretion of pro-inflammatory cytokines such as IL-17 and IL-23 (198). On the other hand, in the early stages of sepsis, CCL1-primarily produced by alveolar macrophages and positively regulated by the Wnt signaling pathway-binds to the CCR8 receptor on gut-derived CD44+Ly6C-IL-7Rhigh CD8low memory γδ T17 cells. This interaction facilitates the migration of these cells from the intestine to the lungs, where they exacerbate pulmonary inflammation by secreting IL-17A (199). However, during the repair phase following lung injury, the Wnt-β-catenin pathway is essential for alveolar epithelial regeneration. A large number of proliferating ATII cells mobilized for repair can activate the pathway via autocrine Wnts, promoting their own proliferation to replenish the damaged epithelium and complete the repair process (200). Additionally, the Wnt-β-catenin signaling pathway influences lung injury repair and fibrotic processes by regulating the differentiation direction of mesenchymal stromal cells (MSCs; historically referred to as mesenchymal stem cells) (201).

### 4.6 The cGAS-STING signaling pathway

The cGAS-STING signaling pathway plays a crucial role in innate immune responses by recognizing pathogenic DNA and initiating antiviral and antimicrobial defenses (202). In ALI, aberrant DNA species—such as mtDNA released from damaged cells and double-stranded DNA within NETs—are recognized and activate cytosolic cGAS. Activated cGAS catalyzes the synthesis of cyclic GMP-AMP (cGAMP) from ATP and GTP. cGAMP then binds to and activates the STING, triggering its translocation from the endoplasmic reticulum to the Golgi apparatus. Subsequently, STING recruits and is phosphorylated by TANK-binding kinase 1 (TBK1) and interferon regulatory factor 3 (IRF3). This signaling cascade ultimately drives type I interferon-and NF-κB-mediated inflammatory responses, exacerbating alveolar septal thickening, increased vascular permeability, and disruption of the pulmonary barrier (203, 204). Furthermore, activation of the cGAS-STING pathway can trigger various forms of regulated cell death, including apoptosis, pyroptosis, autophagy, and ferroptosis (205). For instance, it can induce apoptosis through a positive feedback loop involving damaged DNA-cGAS-STING, which activates the STING-TBK1-IRF3 pathway leading to Caspase-3 activation (206). It can also regulate pyroptosis via the cGAS-STING-NLRP3 signaling axis (207) and modulate ferroptosis by influencing lipid peroxidation, iron metabolism, and the antioxidant system (208). Simultaneously, a bidirectional regulatory relationship exists between this pathway and autophagy. Excessive cGAS-STING activation can block autophagic flux and inhibit autophagic degradation (209). Conversely, autophagy can clear cytoplasmic DNA, thereby mitigating overactivation of the STING pathway and subsequent tissue damage (210).

Sustained cGAS-STING activation also links acute exudative inflammation to the fibroproliferative, maladaptive remodeling phase of ALI mentioned above. Accumulated cell-free DNA including mtDNA and NET-derived DNA drives persistent STING signaling, promoting fibroblast-to-myofibroblast transition and extracellular-matrix deposition, which bridges acute alveolar injury to post-ALI pulmonary fibrosis. Therefore, STING inhibition represents a multi-stage therapeutic target for both acute inflammatory burst and subsequent fibrotic progression. Selective STING small-molecule inhibitors C-176 and H-151 have shown protective effects in pre-clinical ALI and fibrosis models by suppressing STING-TBK1-IRF3-driven inflammatory and pro-fibrotic outputs (211). Repurposing clinically approved drugs offers attractive translational advantages. For instance, fluvoxamine, an FDA-approved SSRI, attenuates bleomycin-induced pulmonary fibrosis via cGAS-STING suppression and is one of the few licensed agents with documented STING-modulating anti-fibrotic activity in lung disease pre-clinical studies (212). This mechanistic background underpins the rationale for STING-inhibitor-loaded nanomedicines described in Section 6.5. Nevertheless, nearly all supporting data come from pre-clinical models; human evidence for STING-directed therapy in ALI and ARDS remain limited.

## 5 Epigenetic modifications

Epigenetics refers to heritable changes in gene expression that occur without alterations to the DNA sequence, determining whether genes are activated or silenced. This process primarily involves histone modifications, DNA methylation, and non-coding RNA mechanisms (213) (**Figure 3**).

**Figure 3:**
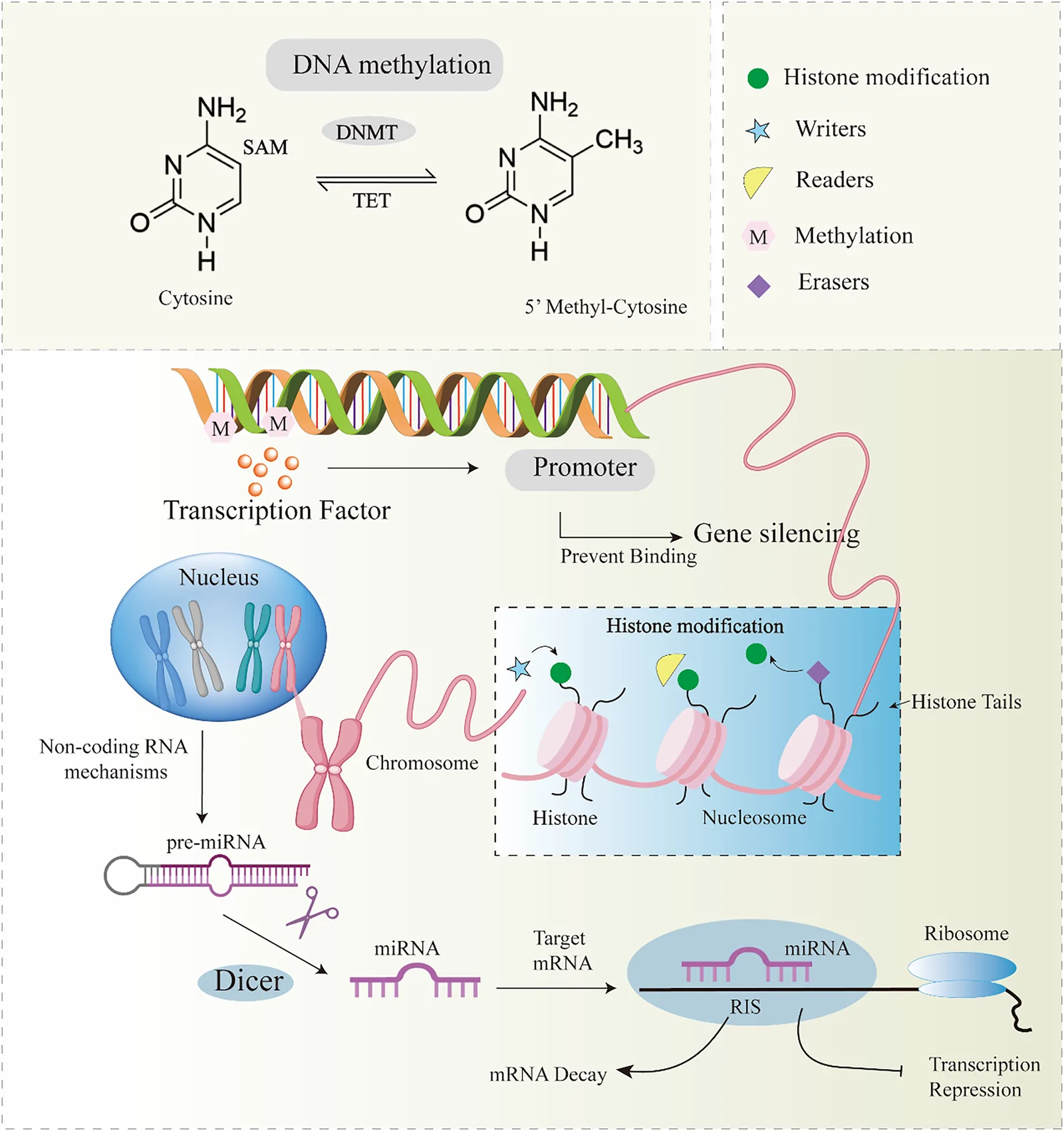
Epigenetic modifications and ALI. Histone modifications, DNA methylation, and non-coding RNA mechanisms are often involved in the development of ALI.

### 5.1 Histone modifications and ALI

Histones are proteins that package DNA into nucleosomes, which are subsequently assembled into chromosomes within the cell nucleus. The N-terminal tails of histones undergo various modifications, including ubiquitination, acetylation, methylation, phosphorylation, deamidation, and ADP-ribosylation (214). Numerous studies have applied histone modifications to the investigation of various diseases, particularly in cancer research, offering novel perspectives for therapeutic strategies (215).

Studies have shown that in sepsis-induced ALI, aberrantly enhanced aerobic glycolysis in lung tissue leads to substantial lactate accumulation. On one hand, the accumulated lactate promotes histone acetyltransferase P300 to catalyze histone H3 lysine 18 lactylation (H3K18la), thereby enhancing the transcription of early growth response protein 1 (EGR1). On the other hand, lactate, mediated by the GNAT domain of histone acetyltransferase KAT2B, induces lactylation at the K364 site of the EGR1 protein. This modification strengthens the binding affinity of EGR1 to importin-α, facilitating its nuclear localization. The activated nuclear EGR1 subsequently upregulates the transcription and expression of heparanase (HPSE). HPSE specifically hydrolyzes heparan sulfate proteoglycans, a core component of the endothelial glycocalyx on pulmonary endothelial cells. This degradation disrupts the glycocalyx structure, resulting in increased pulmonary vascular permeability and ultimately exacerbating inflammatory infiltration and lung tissue injury (216, 217). Additionally, H3K18la can specifically bind to the METTL3 promoter, enhancing its N6-methyladenosine modification activity. This promotes the expression of ACSL4 in alveolar epithelial cells, aggravating ferroptosis-associated lung injury (218).

In LPS-induced ALI, the activated TLR4/NF-κB pathway can induce Jumonji C family of histone 3 demethylases (JMJD3) to demethylate histone H3 lysine 27 trimethylation (H3K27me3) (219), thereby upregulating the expression of C/EBPβ. As a transcription factor, C/EBPβ binds to the promoter of the H3K4 demethylase KDM5A, upregulating its expression. KDM5A subsequently catalyzes the demethylation of histone H3 lysine 4 trimethylation (220), which inhibits the transcription of the anti-inflammatory gene IL-4-induced gene-1 (221). This cascade ultimately increases the M1/M2 ratio and exacerbates ALI (222). Additional studies indicate that in sepsis-induced ALI, histone methyltransferase enhancer of zeste homolog 2 suppresses the expression of ubiquitin-specific protease 10 (USP10) by promoting H3K27me3 modification on the USP10 promoter. This inhibition enhances the ubiquitination and degradation of glutathione peroxidase 4, leading to intracellular ROS accumulation, glutathione depletion, and iron overload. Consequently, this promotes ferroptosis in alveolar epithelial cells, intensifying pulmonary inflammatory infiltration and pathological injury (223). Furthermore, the acetylation of H3K27 also plays a significant role in ALI. In macrophages, activated histone deacetylase 3 removes acetyl groups from H3K27, inhibiting the transcriptional expression of autophagy-related gene 5. This results in impaired autophagic flux and ROS accumulation, which subsequently activates NLRP3 inflammasome assembly (224).

### 5.2 DNA methylation and ALI

In the pathogenesis of ALI, DNA methylation serves as a key epigenetic regulatory mechanism, extensively involved in inflammatory responses, imbalanced gene expression, and cellular dysfunction through differential methylation patterns. This process is primarily catalyzed by DNA methyltransferases, which utilize S-adenosylmethionine (SAM) as the methyl donor to convert cytosine to 5-methylcytosine at CpG island regions, particularly within gene promoters, thereby modulating transcriptional activity (225). Studies have shown that different pathogenic factors can participate in the pathological process of ALI through the differential regulation of DNA methylation (226). In a rat model of LPS-induced ALI, genome-wide methylation analysis revealed aberrant methylation involving 1,721 genes in promoter regions and 990 genes in CpG island regions. These genes are enriched on chromosomes 1, 3, 5, 7, and 10. By comparing methylation levels and CpG density, genes associated with high-CpG-density promoters were found to have a higher proportion of methylation. The aberrant methylation primarily targets the regulation of the MAPK signaling pathway, and inflammation-related genes such as Cebpb, Cxcl2, IL-6, Mylk2, and Mif exhibit abnormal DNA methylation patterns (227). Mechanistically, LPS can activate the deubiquitinase USP30 in endothelial cells. USP30 stabilizes methionine adenosyltransferase 2A via deubiquitination, promoting SAM synthesis. As the crucial methyl donor, the increased SAM elevates global DNA methylation levels by approximately 35%. This hypermethylation suppresses the transcriptional expression of the anti-inflammatory regulator miR-30a-5p, leading to increased levels of its pro-inflammatory targets MDM2 and NFAT5. Consequently, this exacerbates endothelial cell inflammation and vascular hyperpermeability, aggravating pulmonary edema and inflammatory infiltration (228).

Furthermore, other environmental factors such as PM2.5 can exacerbate lung injury by disrupting methylation homeostasis. PM2.5 exposure leads to downregulated expression of DNMT1 and upregulated expression of DNMT3b in rat lung tissue, while also reducing the plasma ratio of SAM to S-adenosyl-L-homocysteine. This disturbance triggers genome-wide hypomethylation and specific methylation abnormalities in inflammation-related genes: hypomethylation of the IL-4 gene promoter elevates its expression, whereas hypermethylation of the IFN-γ gene promoter suppresses its expression. Consequently, the Th1/Th2 balance is disrupted, intensifying inflammatory infiltration in lung tissue (229). On the other hand, in certain pathological contexts such as viral pneumonia, maintenance DNA methylation mediated by the epigenetic regulator ubiquitin-like with PHD and RING finger domains plays a critical role in the reparative function of induced regulatory T cells. This highlights that dynamic methylation equilibrium is also indispensable for pulmonary immune regulation (230).

### 5.3 MicroRNAs and ALI

MicroRNAs (miRNAs) are a class of highly conserved single-stranded RNAs, approximately 22 nucleotides in length. They regulate gene expression by binding to the 3’-untranslated region of target mRNAs, leading to the prevention of translation or promotion of mRNA degradation (231). These molecules play critical roles in core pathological processes of ALI, including inflammatory responses, cellular function regulation, and tissue repair (232), and are closely associated with intercellular communication. To date, the human genome is known to encode over 2,000 mature miRNAs (233).

miRNAs participate in the dysregulated inflammatory response of ALI through a bidirectional regulatory effect. Among them, injury-associated miRNAs primarily exacerbate inflammation by activating the NF-κB signaling pathway. For example, miR-34a, miR-326, miR-300, and miR-155 target Forkhead box O3 (234), B-cell lymphoma 2-related A1 (235), IκBα protein (236), and suppressor of cytokine signaling 1 (SOCS1) (237), respectively, collectively contributing to NF-κB pathway activation. Additionally, miR-1246 activates oxidative stress, inflammatory responses, and endothelial cell apoptosis by targeting the angiotensin-converting enzyme 2 (ACE2) gene (238). On the other hand, protective miRNAs mitigate ALI progression by acting on various signaling pathways. For instance, miR-223 (239), miR-802 (240), miR-142 (241) can inhibit ALI development by negatively regulating the NLRP3 inflammasome. miRNAs such asmiR-27a (242), miR-16 (243), miR-145-5p (244), miR-182 (245), miR-140-5p (246), miR-128 (247) suppress the downstream TLR4/MyD88/NF-κB signaling pathway by targeting the 3′-UTR of TLR4 mRNA. Meanwhile, miR-21 (248), miR-216a (249), miR-30b5p (250) can inhibit the JAK2/STAT3 signaling pathway. Furthermore, miR-21a-3p promotes lung tissue repair and angiogenesis by modulating the PI3K (p110α)/Akt/mTOR pathway (251). miR-454 inhibits the production of inflammatory cytokines by downregulating CXCL12 mRNA translation (252). Lastly, miR-339-3p inhibits the AKT/mTOR pathway by targeting annexin A3 (253).

Mitochondrial dysfunction, manifested as impaired energy metabolism, excessive generation of mtROS, and imbalances in fission/fusion, directly exacerbates lung injury. Meanwhile, miRNAs can precisely regulate this pathological process by targeting mitochondrial-related pathways (231).On one hand, miRNAs can shuttle between cells via carriers such as extracellular vesicles (EVs) and influence mitochondrial quality control by targeting specific genes. For instance, miR-223, derived from Ly6G+ neutrophils, reduces NLRP3 inflammasome activity, thereby alleviating ALI induced by mitochondrial DAMPs (239). Similarly, miR-21 exerts protective effects against lung injury by elevating mitochondrial membrane potential and reducing the expression of fission proteins Drp1 and Fis1 (254). On the other hand, mtDNA released from damaged mitochondria can act as a DAMP to activate TLR9 signaling, which in turn modulates miRNA expression, forming an inflammation-amplifying loop. For example, miR-34a-5p promotes endothelial dysfunction and mitochondrial-mediated apoptosis in murine models of ALI by downregulating its target gene SIRT1. This facilitates the translocation of p53 and Bax to mitochondria, disrupts mitochondrial membrane potential, triggers cytochrome C release, and ultimately induces mitochondrial-dependent apoptosis in lung cells (255). This bidirectional regulatory network between mitochondria and miRNAs integrates critical pathological processes such as metabolic dysregulation, inflammatory activation, and cell death, profoundly influencing the progression of ALI. It thereby provides novel molecular targets for potential clinical intervention.

## 6 Treatment for ALI

Although significant breakthroughs have been achieved in understanding the pathophysiological mechanisms of ALI, effective strategies for its prevention and treatment remain limited. The core pathological process involves the accumulation of protein-rich exudate containing inflammatory cells within the alveolar spaces and lung interstitium, which triggers inflammation, damages lung tissue, and ultimately leads to increased capillary permeability and respiratory failure. Therefore, intervention strategies focusing on maintaining pulmonary oxygenation, clearing pulmonary edema, suppressing the inflammatory response, and restoring the integrity of the pulmonary barrier are crucial. Currently, a multi-dimensional intervention framework has been established, with lung-protective ventilation and fluid management as the foundation, and targeted pharmacotherapy, mesenchymal stromal cell therapy, and nanomaterial-based delivery technologies as its core components (77).

### 6.1 Lung protective ventilation

Lung-protective ventilation (LPV) is a supportive therapy that can improve survival rates. Its core strategy is based on low tidal volume ventilation, with the physiological rationale of reducing driving pressure (ΔP; defined as plateau pressure minus total-PEEP, representing the effective pressure distending lung parenchyma), mechanical power, and the risk of volutrauma (256, 257), while also mitigating systemic inflammatory responses (258). The landmark ARDS Network trial led to the recommendation of a tidal volume (*V*_T_) of 6 mL/kg predicted body weight (PBW) as the standard for ARDS management (259–261). Positive end-expiratory pressure (PEEP) refers to the pressure maintained at the end of exhalation during mechanical ventilation. However, these studies have inherent limitations. Firstly, they focus solely on comparisons between polar extremes of tidal volume and fail to directly validate the relative merits of intermediate tidal volumes in the range of 7–11 mL/kg (262, 263). Secondly, confounding factors in the high tidal volume groups—such as unaddressed airway pressures—and abnormally elevated respiratory rates in the low tidal volume groups may have exaggerated the absolute benefit of a fixed low tidal volume (264). In clinical practice, adherence to the 6 mL/kg tidal volume target remains low, at only 20%-39% (265). Therefore, to ensure the safety and efficacy of mechanical ventilation, individualized assessment and a clinically judicious approach are imperative. Modern protective-ventilation concepts extend beyond simple tidal-volume limitation, aiming to constrain regional stress-strain and cumulative mechanical power, mitigate atelectrauma, and recognize mechanical ventilation not merely as gas-exchange support but also as a biological modifier of pulmonary inflammation and cellular injury, while simultaneously screening for excessive spontaneous inspiratory effort to reduce risk of P-SILI.

PEEP titration is another critical component of lung-protective ventilation, serving to open collapsed alveoli, improve ventilation distribution, and reduce alveolar overdistension. Guidelines recommend a higher PEEP strategy for patients with moderate-to-severe ARDS, which can significantly improve oxygenation and prognosis, particularly in PEEP-responsive patients (defined by an increase in the P/F ratio of >25 mmHg) (266). Multiple bedside tools including electrical impedance tomography and esophageal pressure monitoring can be used to individualize PEEP settings, though these modalities are not universally available in routine clinical practice. Consistent with the 2024 official American Thoracic Society (ATS) clinical practice guideline update, for patients with moderate-to-severe ARDS, higher PEEP strategy (without routine lung-recruitment maneuvers) is conditionally recommended, while prolonged recruitment maneuvers are not advised for this population (267). Higher-PEEP strategy is not applicable for mild ARDS patients, and clinical implementation still requires individualized titration guided by oxygenation and respiratory mechanics (268).

Prone positioning represents one of the few supportive interventions proven to reduce mortality in moderate-to-severe ARDS. The landmark PROSEVA multicenter randomized controlled trial demonstrated that early prolonged prone positioning (≥16 hours per session) in patients with moderate-severe ARDS (PaO_2_/FiO_2_<150 mmHg with PEEP ≥5 cm H_2_O) significantly reduced 28-day and 90-day mortality compared with supine ventilation, when applied on top of standard lung-protective ventilation (269). Physiologically, prone positioning homogenizes regional lung aeration, reduces transpulmonary stress-strain, improves ventilation-perfusion matching and facilitates airway secretion clearance, thereby mitigating ventilator-induced lung injury. Current guidelines conditionally recommend early prone positioning for mechanically ventilated patients with PaO_2_/FiO_2_ < 150 mmHg. It should be implemented with careful attention to pressure-related injury risk, and adequate staffing is required for safe patient turning and monitoring.

Furthermore, the regulation of driving pressure (ΔP) and mechanical power represents an extended optimization strategy for lung-protective ventilation. Driving pressure (ΔP), identified as a mortality predictor independent of tidal volume, should be strictly maintained below 15 cm H_2_O (270). A pooled analysis of 3562 patients by Amato et al. confirmed that a reduction in driving pressure (ΔP) is closely associated with improved survival. This benefit from lowering tidal volume to reduce driving pressure (ΔP) is particularly significant in patients with low lung compliance (271). Meanwhile, mechanical power should be kept below 17 J/min (161). Its core determinants are driving pressure (ΔP) and respiratory rate, which can be clinically managed by reducing tidal volume, optimizing PEEP, or controlling the respiratory frequency.

Lung-protective ventilation in specific clinical scenarios requires individualized adaptation. For early ALI patients receiving non-invasive ventilation, such as high-flow nasal cannula oxygen therapy, vigilance is required against disease progression due to insufficient ventilatory support, with timely transition to invasive ventilation and adherence to LPV principles when necessary (272). For patients with refractory hypoxemia, salvage modalities such as airway pressure release ventilation (APRV) or high-frequency oscillatory ventilation (HFOV) may be cautiously considered. However, APRV offers limited control over tidal volume, and HFOV may only benefit patients with severe ARDS (defined as a PaO_2_/FiO_2_ratio ≤100 mmHg), potentially increasing mortality in those with mild-to-moderate disease, thus necessitating strict patient selection (273). It is important to note that while recruitment maneuvers can transiently improve oxygenation, they confer no clear mortality benefit and may induce complications such as hypotension and arrhythmias. Their routine use is not recommended; they may only be considered in select patients with significant atelectasis using a gentle, stepwise incremental then decremental PEEP titration approach (274). Consistent with the 2024 official American Thoracic Society (ATS) clinical practice guideline update, for patients with moderate-to-severe ARDS, higher PEEP strategy (without routine lung-recruitment maneuvers) is conditionally recommended, while prolonged recruitment maneuvers are not advised for this population. It should be emphasized that higher-PEEP strategy is not applicable for mild ARDS patients, and clinical implementation still requires individualized titration guided by oxygenation, respiratory mechanics and monitoring tools such as electrical impedance tomography or esophageal manometry (22).

Such patient-level biological heterogeneity also imposes practical considerations for respiratory support and overall clinical management. Conventional stratification schemes, such as Berlin severity classification and the distinction between direct- versus-indirect lung injury, provide useful clinical reference but have limited predictive value for individual treatment responses. The biomarker-defined hyper- and hypoinflammatory subphenotypes show promising capacity to forecast therapeutic effects. Patients falling into the hyperinflammatory subgroup may derive greater benefit from conservative fluid strategies and strict lung-protective ventilation settings, while patients with hypoinflammatory features can often tolerate alternative ventilation and fluid regimens (275). It should be noted that these subphenotypes were identified from retrospective latent-class analysis of historical trial datasets. Robust, simple-to-use bedside tools for rapid subgroup assignment are still lacking, and prospective clinical trials evaluating therapies tailored to these subphenotypes remain scarce. Consistent with ESICM guideline recommendations, further research is required to translate multi-dimensional phenotyping concepts into daily bedside clinical practice.

### 6.2 Fluid management

The core objective of fluid management is to precisely regulate fluid balance, mitigate injury to the alveolar-capillary barrier, and clear pulmonary edema, thereby optimizing ventilation-perfusion matching and pulmonary functional reserve. This strategy, supported by high-level evidence, is a key intervention for improving patient outcomes. The landmark ARDS Network trial demonstrated that compared to a “liberal” strategy, a “conservative” strategy (targeting a central venous pressure < 4 mmHg and a pulmonary artery wedge pressure < 8 mmHg), while not significantly reducing overall mortality, effectively reduced fluid balance, leading to significantly increased ventilator-free days and days out of the ICU (276). Clinical practice necessitates individualized selection of resuscitation fluids based on etiology: colloids are preferred for ALI related to severe hemorrhagic trauma to maintain intravascular volume stability, whereas crystalloids remain the first-line choice for septic shock complicated by ALI. Patients with massive blood loss require timely blood product replacement. Strict control of infusion rates is crucial to avoid aggravating lung injury through excessive tissue edema from overly rapid administration or compromising circulatory stability due to overly slow infusion (77).

The precise implementation of fluid management relies on the rational application of dynamic monitoring techniques. Point-of-care ultrasound serves as a non-invasive monitoring tool to assess venous congestion by evaluating inferior vena cava diameter and collapsibility, respiratory variation, and hepatic vein S-wave reversal (277, 278). This can be combined with assessing the respiratory variation of the left ventricular outflow tract velocity-time integral (a variation ≥15-20% suggests fluid responsiveness) to guide fluid adjustment (279). This multimodal approach helps overcome the limitations of traditional central venous pressure monitoring, which has limited predictive value in critically ill ALI patients. Furthermore, integrating the E/E’ ratio (where >15 suggests elevated left ventricular filling pressure and <8 suggests normal) with lung ultrasound assessment can significantly enhance the accuracy of volume status evaluation (280, 281). It is crucial to recognize that fluid management must strike a balance between lung protection and circulatory stability. Overly restrictive fluid administration may lead to inadequate tissue perfusion, while fluid overload exacerbates pulmonary edema and lung injury. This balance is particularly critical for ALI patients complicated by acute cor pulmonale, where meticulous volume regulation is required to avoid increasing right ventricular strain. For patients supported by venovenous extracorporeal membrane oxygenation (V-V ECMO), a conservative fluid strategy remains applicable and can support the ECMO goal of “lung rest” by reducing pulmonary exudation and the risk of ventilator-induced injury (282).

Current fluid management still faces numerous challenges, such as the propensity of crystalloids to extravasate into the interstitial space, leading to inadequate volume expansion effect; the higher cost of colloids and their potential to induce coagulopathy or allergic reactions; and the risk of immunologic reactions associated with blood products. The combined application of diuretics and albumin can shorten the duration of mechanical ventilation and mitigate pulmonary inflammatory responses. However, caution is warranted regarding the potential decrease in cardiac output and inadequate pulmonary perfusion that may result from diuretic use (283).

### 6.3 Mesenchymal stromal cell therapy

MSCs are a type of adult stem cell characterized by their multipotent differentiation capacity, immunomodulatory activity, and tissue repair potential. They can be isolated from a wide variety of sources, including bone marrow, adipose tissue, umbilical cord, and placenta (284). Furthermore, they adhere to the minimal defining criteria established by the International Society for Cellular Therapy, which include adherent growth, expression of specific surface markers, and the potential to differentiate into osteoblasts, adipocytes, and chondroblasts (285). In the pathological progression of ALI, MSCs intervene in disease development through both direct actions and paracrine effects, targeting multiple dimensions such as antibacterial and antiviral defense, anti-inflammation, tissue repair, and the regulation of apoptosis and oxidative stress (286).

MSCs secrete antimicrobial peptides such as cathelicidin, defensins, and elafin, which exert antibacterial effects by inhibiting bacterial DNA/RNA synthesis (287). The cleavage product of cathelicidin, LL-37, exhibits broad-spectrum antimicrobial activity against both Gram-negative and Gram-positive bacteria, and can also enhance the host’s resistance to viruses by modulating the TLR signaling pathway (288). Furthermore, MSCs secrete interferon-stimulated genes (e.g., CCL2, IFI6, ISG15, PMAIP1, SAT1, and p21/CDKN1A), which can bolster cellular resistance to infections by various viruses such as Dengue virus, Ebola virus, Influenza A virus, and West Nile virus (289), thereby promoting viral clearance and reducing virus-induced lung injury.

The keratinocyte growth factor secreted by MSCs can upregulate the expression and function of the epithelial sodium channel in AT2 cells via the Gab1/ERK/NF-κB signaling axis, thereby enhancing alveolar fluid clearance, alleviating pulmonary edema, and reducing lung tissue inflammatory injury (290). Additionally, MSCs exert therapeutic effects by precisely modulating the function of recruited mononuclear phagocyte (MNP) subsets in ALI lung tissue. Specifically, they inhibit the polarization of Ly6C^hi^CD38^+^ monocytes toward M1 macrophages and reduce neutrophil chemokine secretion. Simultaneously, they block the differentiation of both Ly6C^hi^CD38^+^ and Ly6C^low^CD38^+^ monocytes into CD38^+^ monocyte-derived dendritic cells and CD11b^+^CD38^+^ dendritic cells, respectively, thereby suppressing antigen presentation and excessive immune responses. This regulatory effect relies on the intervention of MSCs in the differentiation trajectory of MNPs, ultimately mitigating pulmonary inflammatory infiltration and immune-mediated injury (291). Further studies indicate that placental mesenchymal stromal cells (pMSCs) can secrete IL-10 to activate the STAT3 signaling pathway, promoting STAT3 phosphorylation and nuclear translocation, which in turn inhibits NLRP3 inflammasome activation. This mechanism achieves the suppression of M1 macrophage polarization and pro-inflammatory cytokine secretion, ultimately attenuating the inflammatory response and conferring protection in ALI through the IL-10/STAT3/NLRP3 axis (292). In summary, MSCs demonstrate clear anti-inflammatory and tissue-repair potential in the treatment of ALI by regulating inflammatory responses and improving the tissue microenvironment through multiple pathways and targets.

In recent years, mounting evidence suggests that the therapeutic effects mediated by MSCs primarily stem from paracrine mechanisms, which are regulated by cytokines and EVs (293). Among these, extracellular vesicles derived from MSCs (MSC-EVs) exhibit lower risks of immune rejection, tumorigenicity, and pulmonary embolism (294), positioning them as a cutting-edge direction in the treatment of ALI and ARDS. MSC-EVs constitute an umbrella term for heterogeneous secreted membrane-bound particles, which are further sub-classified according to biogenesis and size into apoptotic bodies (>1000 nm, formed during apoptosis), exosomes (30–150 nm, originating from inward endosomal budding within multivesicular bodies), and microvesicles (100-1000 nm, generated by outward plasma-membrane budding) (295). Importantly, exosomes represent only one sub-population of extracellular vesicles; these two terms must not be used interchangeably. Their core mechanisms focus on a triple effect. First, anti-inflammatory and immunomodulatory actions. Studies show that MSC-EVs are enriched with miR-181a-5p, which can be internalized by human monocyte-derived macrophages. This miRNA downregulates phosphatase and tensin homolog expression, activates phosphorylated signal transducer and activator of transcription 5, and subsequently upregulates SOCS1. This axis significantly reduces LPS-induced TNF-α and IL-8 secretion, lowers protein concentration in bronchoalveolar lavage fluid, and reduces neutrophil infiltration (296). Second, barrier protection and tissue repair. Researchers have constructed a biomimetic ALI model based on a microfluidic chip. This model co-cultures human alveolar epithelial cells and lung microvascular endothelial cells on opposite sides of a porous membrane to simulate the alveolar-capillary barrier. LPS induction successfully replicates core pathological features of ALI. MSC-EVs are efficiently internalized by target cells, upregulate the expression levels of tight junction proteins, significantly restore the transepithelial/transendothelial electrical resistance of the damaged barrier, reduce the number of intercellular gaps, inhibit cell death, and promote proliferation. This confirms their therapeutic role in ALI by restoring alveolar-capillary barrier integrity (297). Third, metabolic reprogramming. Exosomes derived from adipose-derived MSCs transfer mitochondrial components (primarily mtDNA) to alveolar macrophages. This transfer significantly increases mtDNA copy number, mitochondrial membrane potential, OXPHOS activity, and ATP generation in LPS-injured macrophages, alleviates mitochondrial reactive oxygen species stress, mitigates cellular energy depletion, and promotes macrophage polarization from the M1 to the M2 phenotype (298).

In summary, MSCs and their derived EVs offer a highly promising dual therapeutic strategy—encompassing both cellular and acellular approaches—for ALI treatment, owing to their multifaceted anti-inflammatory mechanisms, barrier repair capabilities, and metabolic regulatory functions. However, current research and application still face limitations, including a lack of standardization in large-scale production, incomplete elucidation of EV components and their mechanisms of action, and the need for further validation through large-scale clinical studies. Future efforts should focus on addressing these fundamental issues to advance their clinical translation (299).

Although pre-clinical findings support robust anti-inflammatory, reparative, and antimicrobial properties of MSCs and MSC-derived extracellular vesicles, clinical translation remains incomplete. Completed phase I/II human trials have primarily focused on safety endpoints. Early phase-I dose-escalation START trial demonstrated that intravenous bone-marrow-derived MSCs were well-tolerated in moderate-to-severe ARDS patients without infusion-related serious adverse events, though significant improvements in predefined clinical endpoints were not achieved (300). The ONE-BRIDGE phase II trial evaluating multipotent adult progenitor cells for pneumonia-associated ARDS showed numerical but non-statistically significant improvements in ventilator-free days and survival in the intervention group, with no new safety signals observed (301). Multiple small-sample clinical studies from China and other regions, including trials for COVID-19-related ARDS, confirmed acceptable safety profiles of adipose-derived, umbilical-cord-derived and placental-derived MSCs, but consistent, definitive efficacy signals are still lacking across heterogeneous ARDS populations. At present, large-scale, definitive phase-III randomized controlled trials for classical ARDS are still ongoing; thus, MSC-based cell therapy remains an investigational strategy and is not ready for routine clinical application for ARDS (302).

### 6.4 Venovenous extracorporeal membrane oxygenation

Venovenous extracorporeal membrane oxygenation (VV-ECMO) serves as an important rescue supportive measure for refractory severe ARDS when maximal conventional therapy fails. The landmark EOLIA randomized controlled trial enrolled severe ARDS patients with very severe hypoxemia despite optimized conventional management; patients were randomized to receive early VV-ECMO versus conventional treatment with cross-over ECMO allowed for refractory hypoxemia in the control arm. The trial was stopped early for futility, and the primary analysis based on frequentist statistics did not reach statistical significance for reduction in 60-day mortality (35 % vs 46 %, p=0.09) (303). Nevertheless, an absolute 11 % mortality reduction was observed in the early-ECMO group, and a substantial proportion of control-arm subjects crossed over to receive rescue ECMO, which complicated interpretation of the primary endpoint (303). Subsequent *post-hoc* Bayesian re-analysis of EOLIA trial data demonstrated a high posterior probability (88 %-99 %, dependent on prior assumptions) that early VV-ECMO conferred a survival benefit in severe ARDS patients, challenging the interpretation derived from classic frequentist testing (304). Individual-patient-data meta-analysis combining EOLIA and CESAR datasets further supported potential survival advantages for selected severe ARDS patients treated with VV-ECMO.

The 2024 ATS guideline issued a conditional recommendation for VV-ECMO use in carefully selected severe ARDS patients who fail conventional maximal supportive therapy (including lung-protective ventilation, optimal PEEP adjustment, prone positioning, and short-course neuromuscular blockade) (22). Candidate patients generally present with very severe hypoxaemia or refractory hypercapnia, with reversible underlying disease a key prerequisite for considering ECMO support (17). Early evaluation for transfer to an ECMO-capable center is reasonable when conventional therapies cannot maintain acceptable gas-exchange targets. VV-ECMO remains resource-intensive and is accompanied by multiple procedure-related complications including bleeding, stroke, infection and circuit-related adverse events, and it cannot be regarded as a universal intervention for all severe ARDS cases. Careful patient selection and management in high-volume experienced centres are critical to optimize outcomes.

### 6.5 Pharmacological therapy

Treatment for ALI and its severe form, ARDS, may help alleviate hypoxemia, attenuate pulmonary inflammation, prevent or delay lung tissue injury, reduce the incidence of multiple organ dysfunction, and preserve patients’ respiratory function to the greatest extent possible (10). Although there is currently no specific curative therapy for ALI and ARDS, early pharmacological intervention can reduce the risk of severe complications and slow disease progression (28). It should be emphasized that evidence levels for pharmacological interventions in ALI and ARDS vary substantially. Few agents possess high-grade evidence from multiple replicated multicenter randomized controlled trials for routine clinical use. Approved drugs are largely supportive or repurposed agents; investigational agents include compounds under clinical trial evaluation and pre-clinical candidates, whose findings cannot be directly extrapolated to routine clinical practice (305). Overall, glucocorticoids and neuromuscular blocking agents are the mainstay of clinical pharmacological treatments for ALI and ARDS, while a number of targeted therapeutic strategies against specific signaling pathways are gradually emerging as research hotspots. The main approved drugs, potential agents under clinical trial, and various compounds in preclinical research are summarized in **Table 1**.

**Table 1:** The main approved drugs, potential clinical trial drugs, and pre-clinical drugs for ALI treatment.

Type	Name	Targets/mechanisms	Indications	Evidence level	References
Glucocorticoid	Dexamethasone	Inhibit NF-κB, reduce proinflammatory cytokines, exert anti-inflammatory effects	Moderate to severe COVID-19-associated ARDS	Established clinical therapy	(306)
	Hydrocortisone	Inhibit NF-κB, reduce proinflammatory cytokines, improve lung function	Early stage of sepsis-associated ARDS	Established clinical therapy	(307)
	Methylprednisolone	Low-dose glucocorticoid effect: ameliorate lung injury, boost oxygenation	ALI	Established clinical therapy	(308)
Neuromuscular blocking agent	Cisatracurium	Reduce metabolic demand, improve synchrony, alleviate ventilator-induced injury	Early stage of severe ARDS (within 48 hours of onset)	Established clinical therapy	(309)
	Rocuronium	Enhance alveolar recruitment, improve synchrony, promote oxygenation	Moderate to severe pediatric ARDS	Established clinical therapy	(310)
	Vecuronium	Improve patient-ventilator synchrony, assist mechanical ventilation	Adjunctive therapy for ARDS with mechanical ventilation	Established clinical therapy	(311)
Anti-complement C5a monoclonal antibody	Vilobelimab	Block C5a-receptor binding, inhibit complement-mediated inflammation	COVID-19-associated ARDS requiring invasive mechanical ventilation	Clinical trial	(312)
Selective inhibitor of NLRP3 inflammasome	MCC950	Inhibit NLRP3 inflammasome, reduce pulmonary inflammation	ALI	Animal study	(313)
antidiabetic agent	Buformin	Upregulate autophagy via AMPK, inhibit NLRP3-mediated pyroptosis	Sepsis-induced ALI	Animal study	(314)
MD2 inhibitor	Chalcone derivatives 7w	Bind MD2 Phe residue, block LPS-MD2-TLR4 complex formation	LPS-induced ALI	Animal study	(315)
	Chalcone derivatives 7x	Bind MD2 Phe residue, block LPS-MD2-TLR4 complex formation	LPS-induced ALI	Animal study	(315)
	Chalcone derivatives Compound 20	Bind MD2, block LPS-induced MAPKs/NF-κB pathway activation	LPS-induced ALI	Animal study	(316)
	Bisaryl-1,4-dien-3-one	Block TLR4/MD2 pathway, inhibit sepsis-related lung inflammation	Septic shock, pulmonary inflammation, and ALI	Animal study	(317)
TLR4 antagonist	FP12	Block HMGB1/MD2/TLR4 axis, inhibit innate immune lung injury	Influenza virus-induced fatal lung injury	Animal study	(318)
JAK2 inhibitor	AZD1480	Downregulate JAK2/STAT1, prevent HMGB1 translocation, reduce injury	Ventilator-induced lung injury and ALI	Animal study	(319)
ADAM17/MMP Inhibitor	Apratastat	Inhibit ADAM17, reduce ACE2 shedding and neutrophil infiltration	COVID-19-associated ALI	Animal study	(320)
	TMI-1	Inhibit ADAM17, prevent leukocyte infiltration, improve lung injury	COVID-19-associated ALI	Animal study	(320)
Nox1/Nox4 Inhibitor	GKT137831	Inhibit NADPH oxidase, reduce ROS-mediated lung injury	ALI induced by lung ischemia-reperfusion injury	Animal study	(321)
Antimicrobial peptides (immunomodulators)	RTD-1	Inhibit neutrophil functions, suppress proinflammatory cytokine release	Endotoxin-induced acute lung injury	Animal study	(322)
S100A8/A9 Inhibitor	ABR2575	Block neutrophil-S100A8/A9-TLRs pathway, inhibit inflammation	ALI	Animal study	(323)

#### 6.5.1 Approved drugs

Glucocorticoids represent one of the most controversial yet promising agents for the treatment of ALI and ARDS to date. By inhibiting the NF-κB signaling pathway and reducing the release of proinflammatory cytokines (e.g., TNF-α, IL-1β, IL-6), glucocorticoids exert broad-spectrum anti-inflammatory, antioxidant, pulmonary vasodilatory, and anti-edematous effects (324). Dexamethasone, a long-acting glucocorticoid, when administered intravenously in combination with standard care to patients with moderate to severe COVID-19-associated ARDS, significantly increases the number of ventilator-free days within 28 days compared with standard care alone (306). Hydrocortisone has been shown to improve pulmonary physiological function in the early treatment of sepsis-associated ARDS, albeit without demonstrating a significant survival benefit (307). A multicenter randomized controlled trial by Steinberg et al. in 2006 confirmed that low-dose methylprednisolone improves the oxygenation index and lung compliance in ALI patients and shortens the duration of mechanical ventilation (308). It is important to note that high-dose glucocorticoids are explicitly not recommended for routine use in clinical guidelines due to the increased risks of infection and metabolic disorders (325). The 2024 ATS guideline gives a conditional recommendation for systemic corticosteroid administration in ARDS patients of all severity grades, with moderate-certainty evidence supporting its use; initiation within 14 days of ARDS onset is suggested, while late-phase administration beyond 14-day disease course is not encouraged (1). Of note, accumulating evidence suggests treatment responses to glucocorticoids vary greatly across ARDS subphenotypes: hyper-inflammatory subphenotypes tend to derive greater clinical benefit, whereas hypo-inflammatory patients may face elevated infection risks after steroid exposure (7).

Neuromuscular blocking agents (NMBAs) hold a pivotal position in the management of critically ill patients with ARDS. Cisatracurium is the most widely used benzensulfonate-based NMBA in clinical practice. The landmark ACURASYS trial demonstrated that a 48-hour continuous infusion of cisatracurium initiated in the early phase of severe ARDS (within 48 hours of onset) significantly improves the adjusted 90-day survival rate and increases the duration of ventilator liberation, without elevating the incidence of ICU-acquired weakness (309). However, the subsequent ROSE trial failed to replicate these beneficial outcomes in patients with moderate to severe ARDS receiving a high PEEP strategy (326). In pediatric patients with moderate to severe acute respiratory distress syndrome, a 48-hour continuous infusion of rocuronium has been shown to potentially enhance alveolar recruitment, improve patient-ventilator synchrony, facilitate oxygenation, and reduce ventilator-associated lung injury, though its efficacy in improving mortality remains controversial (310). Vecuronium is commonly used as an NMBA to assist respiratory management during mechanical ventilation for ARDS patients; a randomized controlled trial evaluated daily vecuronium administration for optimizing patient-ventilator synchrony, yet the study found no significant differences in patient outcomes across different ventilation modes (311). The therapeutic effects of NMBAs are thought to be mediated by reducing metabolic demand, improving patient-ventilator synchrony, mitigating ventilator-induced lung injury, and exerting potential anti-inflammatory properties (327). According to the updated 2024 ATS guideline, neuromuscular blocking agents are conditionally suggested only for early-phase severe ARDS patients (onset within 48 h and PaO_2_/FiO_2_≤100 mmHg), with suggested treatment duration limited to no more than 48 h (22). Routine administration of NMBAs is not recommended for patients with moderate ARDS or for patients receiving treatment beyond the early disease window (1).

Notably, no specific pharmacotherapies have been approved exclusively for the treatment of ALI and ARDS to date. The majority of current treatment regimens are primarily supportive and symptomatic, including lung-protective mechanical ventilation strategies (low tidal volume, moderate PEEP), prone positioning ventilation, and conservative fluid management (56). Individualized treatment plans require continuous assessment and timely adjustment based on the patient’s clinical status and treatment response.

In China, sivelestat sodium (neutrophil elastase inhibitor) and ulinastatin (urinary trypsin inhibitor) are widely applied in routine ICU clinical practice for ALI and ARDS management, although high-quality global multi-center randomized evidence supporting mortality benefit remains limited. Sivelestat sodium suppresses neutrophil elastase-mediated pulmonary parenchymal injury, reduces inflammatory mediator release and improves oxygenation in subgroups of ARDS patients, especially sepsis- or COVID-19-associated ARDS (328). Retrospective and domestic prospective cohort studies suggested improvements in oxygenation index and shortening of mechanical-ventilation duration, but consistent reduction in all-cause mortality could not be universally demonstrated across all patient subgroups. Ulinastatin inhibits multiple serine proteases, suppresses excessive neutrophil and macrophage activation, mitigates systemic inflammatory response and organ injury. Meta-analyses of Chinese randomized controlled trials indicated that ulinastatin could improve respiratory parameters and shorten ICU stay, while robust international multicenter validation data are lacking (329). Currently both agents are mainly used according to real-world clinical experience in Chinese intensive-care settings rather than global guideline-level strong recommendations.

#### 6.5.2 Drugs in clinical trials

Excessive complement system activation contributes to immunothrombosis and inflammatory amplification in ARDS, with complement fragment C5a driving neutrophil recruitment, endothelial injury and pro-inflammatory mediator release. Vilobelimab, a monoclonal antibody targeting C5a, blocks C5a-receptor interaction to dampen downstream inflammatory cascades. In the global phase 3 PANAMO trial (NCT04333420), among invasively mechanically ventilated patients with COVID-19-associated ARDS, vilobelimab plus standard-of-care reduced 28-day all-cause mortality compared with placebo (32 % versus 42 %) (312). It has obtained emergency-use authorization in the United States and conditional marketing authorization in the European Union, but its indication is restricted exclusively to COVID-19-related ARDS and it is not approved for non-COVID-19 ARDS populations. Of note, *post-hoc* analyses suggested heterogeneity of treatment response across patient subgroups, and independent validation in non-COVID-19 ARDS cohorts remains absent (330).

Multiple other pharmacologic candidates have entered phase 2-3 clinical testing for ARDS, though robust positive primary endpoints are still lacking. Baricitinib, a JAK-1/2 inhibitor, demonstrated signal of clinical benefit in observational cohorts and retrospective analyses for COVID-19-associated ARDS; however, definitive randomized trial evidence for generalized ARDS remains insufficient (331). Ulinastatin has undergone phase-2 clinical evaluation in ARDS populations in Asia, but large-scale multinational randomized controlled data confirming mortality benefit are still absent (332). Many additional agents (including statins, inhaled prostacyclin analogs, and anti-angiopoietin-2 antibodies) have failed to meet primary clinical endpoints in completed large-scale ARDS trials, highlighting substantial translational barriers from pre-clinical findings to patient outcomes.

#### 6.5.3 Drugs in pre-clinical studies

The NLRP3 inflammasome plays a pivotal role in the pathogenesis of ALI by regulating pyroptosis—a highly inflammatory form of programmed cell death—and thereby exacerbating pulmonary inflammatory injury (333). NLRP3-mediated pyroptosis induces alveolar epithelial cell damage, increased pulmonary microvascular endothelial permeability, excessive activation of alveolar macrophages, and neutrophil dysfunction, which collectively drive the progression of ALI (333). Targeting the NLRP3 inflammasome and pyroptotic pathway has therefore emerged as a promising therapeutic strategy for ALI. A variety of NLRP3 inhibitors have exhibited significant anti-inflammatory and tissue-protective effects in preclinical models. MCC950, a selective NLRP3 inhibitor, can markedly attenuate pulmonary inflammatory responses and cytokine production (313). Buformin, an antidiabetic agent, upregulates autophagy and increases Nrf2 protein levels via an AMPK-dependent pathway, thereby inhibiting NLRP3-mediated macrophage pyroptosis and exerting both therapeutic and preventive effects in sepsis-induced ALI (314).

Myeloid differentiation protein 2 (MD2), a co-receptor of TLR4, plays a critical role in mediating LPS-induced ALI (315). MD2 inhibition, either via pharmacological inhibitors or genetic knockout, can significantly alleviate ALI in animal models, rendering it an attractive therapeutic target for ALI (315). Chalcone derivatives 7w and 7x have been identified as novel MD2 inhibitors that bind directly to the phenylalanine (Phe) residues in the hydrophobic pocket of MD2, blocking the formation of the LPS-MD2-TLR4 complex (315). In a murine model of ALI, oral administration of 7w or 7x significantly attenuated LPS-induced lung injury, pulmonary edema, increased vascular permeability, and inflammatory cell infiltration (315). Another chalcone derivative (Compound 20) binds to the hydrophobic pocket of MD2 via hydrogen bond formation with Arg90 and Tyr102 residues of MD2, effectively blocking LPS-induced activation of the downstream proinflammatory MAPKs/NF-κB signaling pathway of the TLR4/MD2 axis and exerting significant protective effects in a rat model of ALI (316). Bisaryl-1,4-dien-3-one, as an MD2 inhibitor, confers marked protective effects against septic shock and pulmonary inflammation (317). FP12, a TLR4 antagonist, can block the HMGB1/MD2/TLR4 axis and protect mice from lethal influenza virus-induced lung injury (318).

The JAK2/STAT1 signaling pathway is a common pathway contributing to a variety of inflammatory diseases, including different forms of ALI (319). In a model of ventilator-induced lung injury, AZD1480, a JAK2 inhibitor, significantly attenuates lung damage and inhibits protein leakage and cytokine release by downregulating the phosphorylation levels of JAK2/STAT1 and preventing the translocation of HMGB1 from the nucleus to the cytoplasm (319). In studies of COVID-19-associated ALI, apratastat and TMI-1, dual ADAM17/MMP inhibitors, have been shown to improve lung histology and prevent leukocyte infiltration (320). Coronaviruses can induce ADAM17 activation, leading to the shedding of ACE2 from the cell surface and thus exacerbating inflammatory responses; accordingly, ADAM17 inhibition can reduce the expression of endothelial adhesion molecules such as ICAM-1 and VCAM-1, and significantly decrease the neutrophil-to-lymphocyte ratio (320).

ROS generated by NADPH oxidase (Nox) may play a key role in the pathophysiological changes of lung ischemia-reperfusion injury (LIRI) (321). GKT137831, a dual Nox1/Nox4 inhibitor, exerts significant pulmonary protective effects in a murine model of LIRI (321).

Rhesus θ-defensin-1 (RTD-1) is an antimicrobial peptide with immunomodulatory activity (334). *In vitro* studies have demonstrated that RTD-1 can inhibit CXCL8-induced neutrophil chemotaxis, TNF-mediated neutrophil-endothelial cell adhesion, and the release of proinflammatory cytokines from activated alveolar macrophages (322). ABR2575, an S100A8/A9 inhibitor, can effectively block the neutrophil-S100A8/A9-TLRs-inflammasome signaling pathway (323). When combined with SiH nanosheets encapsulated in poly(lactic-co-glycolic acid) (PLGA) nanosponges, this agent enables the sustained release of hydrogen to alleviate ROS-induced lung tissue damage and promote M2 polarization of macrophages (323). This novel combinatorial strategy markedly inhibits the infiltration of neutrophils and proinflammatory macrophages into the lung.

#### 6.5.4 Cutting-edge technology therapy

Surfactant-based drug delivery represents an emerging therapeutic strategy. Pulmonary surfactant can serve as a natural drug carrier, and its inherent affinity for lung tissue enhances the distribution of therapeutic agents in damaged pulmonary regions. Preclinical studies have shown that methotrexate-loaded PLGA nanoparticles combined with near-infrared irradiation exert sustained and superior therapeutic effects in arthritis inhibition, a strategy that may also be applicable for the targeted treatment of ALI (335).

The CRISPR-Cas9 gene editing technology has opened up new possibilities for gene therapy of ALI and ARDS. Researchers have utilized this technology to convert induced pluripotent stem cells into “smart” MSCs, which are engineered with a synthetic gene circuit regulated by IL-1. These cells can autonomously secrete therapeutic levels of the IL-1 receptor antagonist upon detecting elevated endogenous IL-1 levels (336). This “sense-and-respond” strategy may provide a novel approach for the personalized and autonomous treatment of ALI.

Despite the persisting therapeutic challenges in ALI and ARDS management, the development of targeted agents against specific signaling pathways and the implementation of refined therapeutic strategies have rendered this condition a generally manageable critical illness. Symptomatic relief can be achieved in numerous patients through the combination of different pharmacotherapies and therapeutic approaches. However, a considerable proportion of patients exhibit poor responses to current treatments, which underscores the necessity of developing novel drugs and therapeutic strategies. It is anticipated that in the near future, several preclinical agents and strategies will successfully advance to clinical research phases, thereby providing more treatment options for patients with ALI and ARDS.

### 6.6 Nanomedicine

Nanoparticles have garnered extensive attention due to their high cell-targeting specificity, biocompatibility, controllable biological activity, and excellent pharmacokinetics (337). In recent years, significant breakthroughs have been achieved with the development of various nanocarriers, including lipid-based, polymer-based, inorganic, and biomimetic types, as well as novel nanoformulations such as nanozymes and nanovaccines. These advancements in regulating inflammatory responses, repairing the pulmonary barrier, and clearing damaging factors have laid a solid foundation for the precise treatment of ALI (338).

Lipid-based carriers, such as liposomes and nanostructured lipid carriers (NLCs), exhibit excellent biocompatibility and membrane fusion properties, enabling the co-loading of both hydrophilic and lipophilic drugs (339). Encapsulating lipophilic antioxidants (e.g., α-tocopherol) and hydrophilic anti-inflammatory agents (e.g., glutathione or dexamethasone) within liposomes has demonstrated significant therapeutic benefits in treating ALI (340). Targeted modification further enhances their specificity; for instance, liposomes decorated with a surfactant protein A (SP-A) nanobody and loaded with methylprednisolone can specifically bind to SP-A, which is highly expressed on alveolar type II epithelial cells. This approach increases drug concentration in lung tissue while significantly reducing systemic side effects (341). NLCs, as second-generation lipid carriers, offer higher drug loading capacity and controlled release characteristics (342). For example, simvastatin-loaded NLCs modified with ICAM-1 antibody can synergistically protect the pulmonary endothelial barrier, reducing vascular leakage and inflammatory infiltration (343). Similarly, melatonin -loaded NLCs conjugated with anti-VCAM-1 antibody alleviate pulmonary inflammation by upregulating Sirtuin 1 to activate NLRP3 inflammasome-dependent pyroptosis (344).

Polymeric nanocarriers, such as PLGA, dendrimers, and self-assembling peptides, enable the co-delivery of drugs and genes to target sites or can function as therapeutic agents themselves for ALI treatment (345). Among these, PLGA is the most commonly used polymer. Studies have shown that PLGA with high biocompatibility, when co-encapsulating fingolimod and nobiletin, exerts anti-inflammatory and antioxidative effects in ALI by inhibiting NF-κB and p38 mitogen-activated protein kinase (346). Dendrimers (e.g., PAMAM, phosphorus dendrimers), with their highly branched three-dimensional structures and abundant surface functional groups, serve as efficient gene carriers (347). For instance, PAMAM-G5 modified with phenylboronic acid and loaded with fibronectin can promote macrophage polarization toward the anti-inflammatory M2 phenotype and reduce the release of TNF-α and IL-1β (348). Curcumin encapsulated within bisphosphonate-modified amphiphilic phosphorus dendron nanomicelles can effectively scavenge ROS and inhibit the regulation of pro-inflammatory cytokines by blocking the NF-κB signaling pathway in M1 alveolar macrophages. This also facilitates the effective repolarization of alveolar macrophages to the anti-inflammatory M2 type, enabling a combination of anti-inflammatory and antioxidative therapy (349). Self-assembling peptide carriers, designed based on specific amino acid sequences, can form nanomicelles or nanofibers to deliver drugs or genes. For example, a nanocomplex formed by the R3V6 peptide, loaded with S1PLyase siRNA and the HMGB1A box A peptide, synergistically blocks the NF-κB pathway to exert anti-inflammatory effects (350).

Inorganic nanoparticles, such as gold nanoparticles (GNPs), cerium dioxide (CeO_2_), and Se@SiO_2_, possess physicochemical properties like enzyme-mimetic activity and photothermal effects, and demonstrate high efficiency in biological applications, exhibiting multifunctional advantages in ALI treatment. Modifying GNPs with peptides is an effective strategy to enhance their efficiency and safety. Studies have shown that a bioactive nanodevice, P12, composed of hexapeptide-coated GNPs, can be administered intratracheally to target pulmonary macrophages. This approach reduces macrophage activation and promotes the transition from the M1 to M2 phenotype (351). A novel class of polydopamine (PDA)-coated CeO_2_nanozyme (Ce@P) enhances ROS scavenging capacity under near-infrared (NIR) irradiation for the treatment of ALI. Ce@P integrates both ROS scavenging and photothermal properties. The PDA coating not only improves the dispersion stability and ROS scavenging efficiency of CeO_2_but NIR irradiation further amplifies its antioxidant and anti-inflammatory effects (352). Porous Se@SiO_2_nanospheres can mitigate paraquat-induced ALI by suppressing ROS, thereby reducing the levels of NF-κB, phospho-NF-κB, and inflammatory cytokines (353).

Biomimetic nanoformulations include cell membrane-camouflaged nanocarriers, cell-derived exosomes/vesicles, and cell hitchhiking delivery systems. Cell membrane-camouflaged nanocarriers involve coating nanocarriers with membranes from cells such as macrophages, neutrophils, or red blood cells, endowing them with innate inflammatory targeting capabilities. For instance, Chengkang Jin’s team constructed a ROS-responsive nanomicelle delivery system camouflaged with a lung epithelial cell membrane. This system employs PLGA-PEG containing a diselenide bond as the carrier for berberine, coated with an MLE-12 lung epithelial cell membrane. Leveraging the homotypic targeting of the cell membrane, the system achieves precise lung accumulation. In the high-ROS microenvironment of ALI inflammation sites, the diselenide bond cleaves, triggering the rapid release of over 80% of the encapsulated berberine while preventing premature drug leakage, thereby enhancing local drug concentration (354). Cell-derived extracellular vesicles utilize naturally secreted nanovesicles (e.g., exosomes derived from mesenchymal stromal cells, platelet-derived extracellular vesicles (PEVs)) as drug carriers. They leverage innate cell-targeting properties and low immunogenicity for efficient drug delivery. Jun Ho Lee’s team isolated adipose-derived mesenchymal stem cell extracellular vesicles (ASC-EVs) from cell culture media via tangential flow filtration and found that ASC-EVs effectively prevent ALI caused by SARS-CoV-2 and H1N1 influenza A virus (355). Kaige Chen’s team developed a polymeric nanoparticle, PC@PLGA, with a PLGA core coated with PEVs and a CRT-overexpressing cell membrane. This design combines the ability to target activated neutrophils and mimic “senescent signals.” P-selectin on the PEVs facilitates the specific anchoring of PC@PLGA to activated neutrophils, while CRT can induce macrophage-mediated programmed cell removal. This allows for the precise clearance of activated neutrophils independent of apoptosis, offering a novel targeted therapeutic strategy for ALI (356). Cell hitchhiking delivery utilizes circulating cells as “vehicles” to transport nanocarriers for lung-targeted delivery. For example, Yaning Ding’s team prepared chitosan nanoparticles (MPSS-CSNPs) loaded with methylprednisolone sodium succinate (MPSS) and adsorbed them onto red blood cell (RBC) surfaces via non-covalent interactions (RBC-MPSS-CSNPs). This delivery system significantly prolongs drug circulation time, reduces uptake by non-target organs such as the liver, achieves precise MPSS enrichment in the lungs, and lowers the peak plasma drug concentration (357).

Novel functional nanotherapeutics include nanozymes, nanovaccines, and metal-organic frameworks (MOFs). Nanozymes are a class of novel nanomaterials with enzyme-like activities, offering advantages such as high catalytic efficiency, functionality under mild reaction conditions, and excellent stability. For example, the inhalable CoAl-layered double hydroxide (CAL) nanosheets developed by Xichun Qin’s team can bind to damaged DNA and effectively suppress the cGAS-STING pathway-mediated inflammatory response. Furthermore, CAL nanosheets can be loaded with the cGAS-STING inhibitor C176, achieving a synergistic effect through dual inhibition of the pathway (203). Nanovaccines utilize nanocarriers to deliver immunogens such as bacterial outer membrane vesicles or antigenic peptides, enabling precise targeting of antigen-presenting cells to activate both specific humoral and cellular immunity. This approach not only clears pathogenic bacteria but also establishes immune memory to prevent reinfection (338). For instance, in a pneumonia model induced by Acinetobacter baumannii, the multiepitope peptide nanovaccine CS-PLGA-rOmp22 induced high levels of specific IgG antibodies and a Th1-type cellular immune response in mice. It significantly reduced the bacterial load in the lungs and blood of infected mice, alleviated lung tissue damage, and provided effective protection against fatal A. baumannii infection (358). MOFs are porous nanomaterials composed of metal ions and organic linkers, characterized by a high surface area, tunable pore size, and stimuli-responsive functionality (338). One study developed a food-grade γ-cyclodextrin metal-organic framework (γCD-MOF) modified with cholesterol (CHS) to load sulfur dioxide (SO_2_) as a dry powder inhaler (DPI) platform (CHS-CD-MOF@SO_2_). This platform targets the lungs for ALI treatment. CHS-CD-MOF@SO_2_reduces the expression of pro-inflammatory factors by modulating the P38 and NF-κB pathways, significantly improving the lung wet/dry weight ratio and reducing neutrophil infiltration. Its therapeutic efficacy was comparable or superior to that of dexamethasone (359).

In summary, the emergence of nanomedicine offers novel strategies to overcome the limitations of traditional pharmaceutical treatments, and nanoparticles are increasingly becoming powerful tools for treating human immune-related diseases. However, significant scientific and translational challenges remain. Future research should focus on further enhancing biocompatibility, refining targeted delivery strategies, optimizing multi-target therapeutic combinations, and conducting comprehensive translational studies.

## 7 Conclusions and prospects

Accumulated pre-clinical and clinical evidence has uncovered multi-layered, highly interconnected pathological cascades underlying ALI and ARDS, encompassing alveolar-capillary barrier disruption, immune dysregulation, organelle stress, mechanical-driven secondary injury, metabolic reprogramming, cellular senescence, as well as aberrant activation of multiple signaling pathways and epigenetic alterations. To synthesize these scattered findings, we provide an integrative conceptual framework that links core pathogenic events with corresponding actionable therapeutic targets (**Figure 4**). Although lung-protective ventilation and conservative fluid management constitute the cornerstone of current clinical supportive care, mechanism-specific targeted therapies that can reliably improve patient outcomes remain largely unmet.

**Figure 4:**
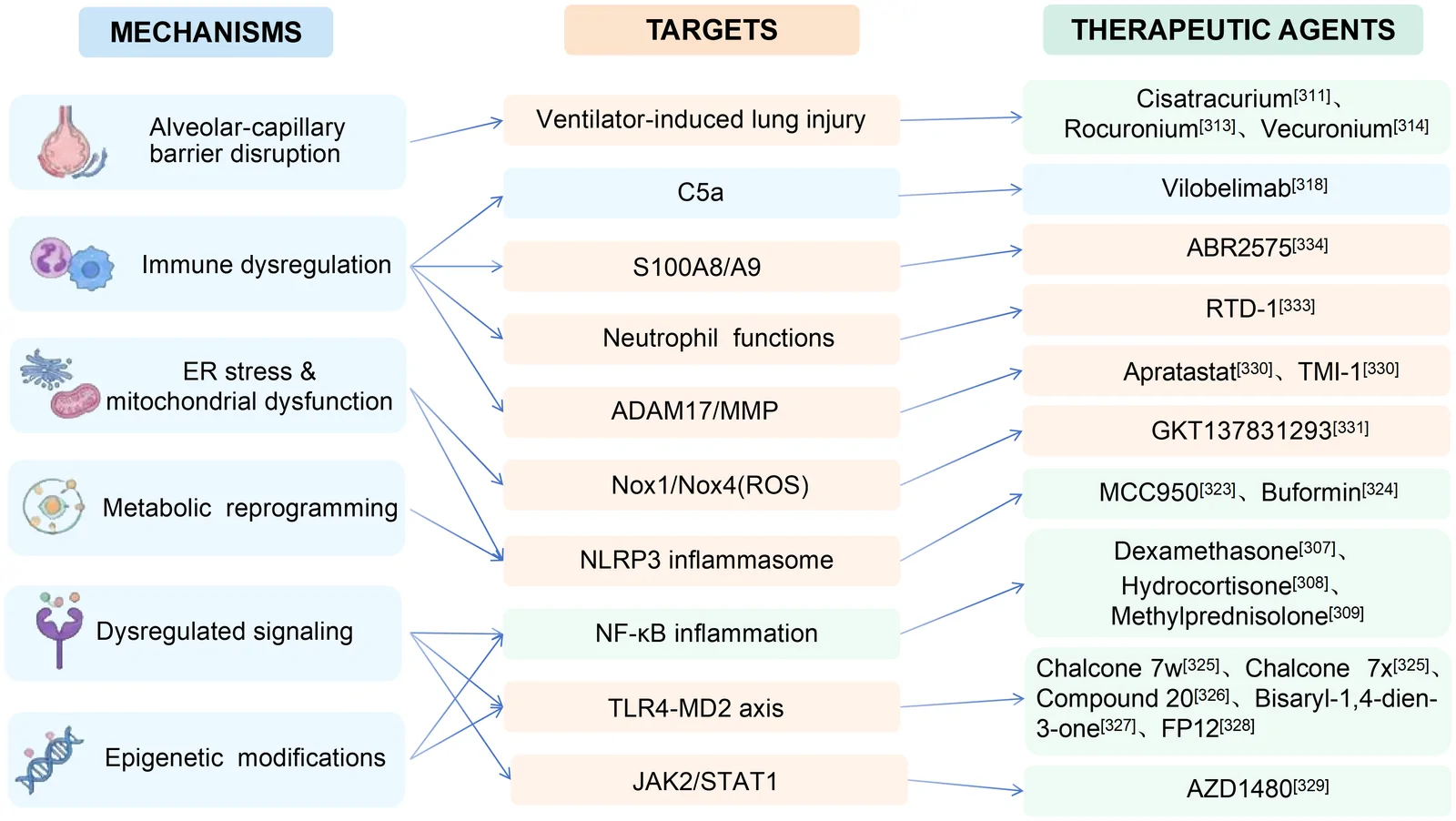
An integrating framework linking major pathological mechanisms and potential therapeutic targets of ALI and ARDS. This schematic summarizes core pathogenic cascades of ALI and ARDS, and maps key pathological nodes to diverse therapeutic targets, including clinical supportive care, pharmacological candidates, cell-based therapy, nanomedicine and epigenetic modulators. It visualizes the complex interconnections among disease mechanisms and provides conceptual reference for developing future precision-oriented therapeutic strategies.

This review systematically examines the pathological mechanisms and therapeutic advances in ALI, a clinically critical syndrome. ALI is characterized by disruption of the alveolar-capillary barrier, involving interrelated pathological processes such as epithelial and endothelial cell injury, excessive infiltration and activation of innate immune cells, endoplasmic reticulum stress and mitochondrial dysfunction, as well as abnormalities in metabolic and epigenetic regulation. Although current supportive therapies centered on lung-protective ventilation and restrictive fluid management have become the clinical standard, targeted interventions that specifically block key disease mechanisms and improve patient outcomes remain lacking. Traditional anti-inflammatory drugs have shown limited success in clinical translation, while emerging strategies such as mesenchymal stem cell therapy and nanomaterial-based delivery systems, though promising in preclinical studies, require further validation regarding their clinical efficacy and safety. In particular, the pulmonary endothelium should be regarded as a central therapeutic target; interventions aimed at preserving glycocalyx integrity, restraining pathological endothelial activation, and mitigating pulmonary microvascular thrombosis may hold great potential to interrupt ALI pathological cascades.

Overcoming the therapeutic bottleneck in ALI hinges on advancing toward individualized precision medicine. This calls for integration of multi-omics data, dynamic biomarker monitoring and clinical profiles, alongside full recognition of substantial biological heterogeneity across human ARDS patients. The hyper-inflammatory and hypo-inflammatory subphenotypes, identified by latent-class analysis from retrospective clinical trial datasets, represent the most well-documented candidate endotypes of ARDS, which exhibit divergent mortality risk and differential responses to fluid strategies, ventilation settings and anti-inflammatory treatments (30). Nevertheless, these subphenotyping approaches still face notable translational barriers: existing classification models rely on multiple plasma biomarkers that cannot be rapidly measured at the bedside, and prospective randomized clinical trials validating subphenotype-tailored therapies are still scarce (360). At present, subphenotyping remains largely confined to research settings rather than routine real-world clinical practice. Developing simple, rapid point-of-care classification tools and conducting prospective interventional trials for subgroup-oriented treatments aligns with ESICM guideline priorities and represents a key promising translational direction for future ALI and ARDS research (29).
